# Automated Optimization of Mixing Elements for Single-Screw Extrusion Using CFD Simulations

**DOI:** 10.3390/polym17040438

**Published:** 2025-02-07

**Authors:** Tanja Matzerath, Christian Bonten

**Affiliations:** Institut für Kunststofftechnik, University of Stuttgart, 70569 Stuttgart, Germany; christian.bonten@ikt.uni-stuttgart.de

**Keywords:** single-screw extrusion, mixing elements, mixing processes, optimization, CFD simulation

## Abstract

Today’s materials must meet high mechanical requirements while remaining cost-effective in production. This requires a homogeneous temperature and material distribution and splitting, as achieved by mixing elements in single-screw extrusion, which depends on the material properties, the geometry of the mixing elements, and the process conditions. Existing computational fluid dynamics (CFD) methods can help, but often optimize dispersive (split) or distributive (spread) mixing separately and neglect their mutual influence and competition, which prevents a single optimal solution. To address this, this work develops an automated optimization tool using a genetic algorithm for the holistic optimization of both mixing processes, considering pressure drop, temperature gradient, and quantitative metrics for dispersive and distributive mixing. Compromises between geometry and metrics that improve dispersive mixing while maintaining moderate temperature gradients and pressure drops were determined for four different polymers. The results of the dispersive mixing element show dependencies between mixing metrics and geometry. In contrast, the distributive mixing element shows no clear correlations between mixing metrics and geometry.

## 1. Introduction

One of the most important criteria in plastics processing is a good melt quality. A good quality can only be achieved with a melt that is homogeneous with regards to its temperature distribution and its material distribution. With the ever-increasing demands on material properties (improved mechanical properties combined with cost-effective production), today’s materials often consist of more than just one polymer. Instead, polymer blends are produced by adding a further component, so-called additives, which can be liquid or solid. The aim of blend production is to combine the properties in an advantageous way and, in the best case, to create synergies [1].

Most polymer blends are usually heterogeneous systems that form a dispersion. It is a system consisting of at least two immiscible components, one component forming the matrix (highly concentrated) and one forming the disperse phase. The morphology of the dispersion describes the shape and distribution of the different components in relation to each other. Since the final properties of a blend material are strongly dependent on its morphology, its structure is of crucial importance [2,3]. The morphology of the blend system is created during the production of a blend. Blends are typically produced using twin-screw extruders, where plastic is melted in a heated barrel with rotating screws, conveying the plastic melt, which is then granulated. In a second step, the granulate is converted into single-screw extruders (SSE) to manufacture end products such as foils, tubes, etc., called extrudates. However, mixing is not only relevant in twin-screw extrusion but also plays a crucial role in many SSE processes. Whether ensuring the uniform incorporation of regrind—where production waste is reintroduced into the material stream—or evenly distributing additives such as color masterbatch, effective mixing is essential for maintaining consistent product quality. In high-performance SSE, characterized by low energy demand and high material throughput, achieving a homogeneous melt presents additional challenges. Due to the high circumferential velocity of the screw, the high viscosity of the melt, and the lack of turbulence in the flow, it is a major challenge for plastics conversion to achieve good material and thermal homogeneity. However, a non-homogeneous melt leads to a decrease in the mechanical properties and thus to a reduction in the quality of the extrudate [1].

To attain the desired homogeneity within the melt, the screw is equipped with dynamic mixing elements. The geometry of these elements significantly impacts the mixing effect in the plastic melt. Two distinct types of mixing elements induce different mixing processes: distributive and dispersive. Dispersive mixing refers to the splitting of the plastic melt into smaller structures, such as the breakup of droplets of the dispersed phase, while distributive mixing involves spreading the material over the domain. Depending on the geometry of the dispersive mixing element, a superposition of elongational and shear flow or only a shear flow can be induced. In a heterogeneous blend, the elongational flow is of crucial importance. It causes the disperse phase to stretch into fibrils which can finally break up into small droplets. With a pure shear flow, there is an upper limit in the shear deformation at high viscosity ratios $\lambda =\frac{\eta_{d}}{\eta_{M}}$, where $\eta_{M}$ is the viscosity of the matrix and $\eta_{d}$ is the viscosity of the disperse phase. For λ>3.5, the shear deformations can no longer break up or deform the disperse phase, regardless of the shear intensity, although the elongational flow can still influence the shape of the disperse phase, even for much higher viscosity ratios [4].

However, this process can result in local aggregation of the disperse phase and high temperature gradients. The distributive mixing element aims to evenly distribute the blend components throughout the extruder cross-section, irrespective of the dispersed phase’s shape, while also ensuring homogeneous temperature distribution within the melt. Therefore, in practice, mixing elements are combined by placing a distributive mixing element after the dispersive element [1]. As these different mixing conditions are mainly controlled by the geometry of the mixing elements, the design of the geometry is of crucial importance. The design is often based on empirical knowledge, which proves to be inefficient in view of the ever-increasing number of new and unknown materials. In addition, the mixing process is associated not only with the mixer geometry, but also with the rheological and thermal properties of the material as well as the operation point such as the circumferential velocity of the screw and the process temperature. The number of dependencies can hardly be taken into account experimentally and would also be a costly undertaking. In addition, conducting experiments is labor-intensive and results are often evaluated visually due to the lack of suitable mixing metrics to objectively quantify the mixing process.

A computational fluid dynamics (CFD) simulation of mixing processes can be used as an alternative cost- and time-saving method. CFD can be used to determine flow fields such as pressure, temperature, and velocity for different computational domains and varying ambient conditions. In doing so, the respective material properties are always taken into account. However, a major challenge is the evaluation of the mixing process in a suitable computational time. Without defined metrics for evaluating mixing effectiveness that can distinguish between good and poor mixing, it is not possible to optimize the mixer geometry and thus the extrusion process. Various approaches already exist that can analyze the mixing process quantitatively. They are alike in that they are based on flow fields previously determined using CFD simulations, but differ in the mathematical evaluations. The approaches can be divided into a kinematic evaluation, as well as statistical and geometrical methods. These methods have certain advantages and disadvantages, particularly with regard to the dispersive and distributive mixing process.

Kinematic evaluation is a well-established technique for evaluating the efficiency of the dispersive mixing process. This method involves the quantification of mixing through the analysis of fluid-element deformations which are induced by prevailing flow dynamics. Within dispersive mixing elements, the fluid is deformed to varying degrees, so that the total deformation must be considered in order to assess the kinematic mixing quality. In prior studies such as [5], the planar elongational rate, the shear rate, and the total deformation at the outlet of a two-dimensional wedge-rectangular slit have been utilized as metrics to quantify dispersive mixing phenomena. These parameters are integrated across the slit height at the mixing element and weighted by volume flow rate. Analysis based on these metrics has revealed that a wedge-rectangular slit induces melt fracture, which facilitates droplet size reduction in the production of heterogeneous polymer blends. Additionally, in [6], a finite fluid element with dimensions $L_{x}$, $L_{y}$, and $L_{z}$ is examined in spatial directions *x*, *y*, and *z*. By evaluating the relative linear elongational ratio $\lambda_{L}=\vec{L}/{\vec{L}}_{0}$, where ${\vec{L}}_{0}$ represents the initial fluid-element size, the extent of dispersive mixing can be inferred. Higher values of $\lambda_{L}$ correspond to greater elongational ratio and enhanced mixing, where $\lambda_{L}=1$ indicates no mixing. This was used to quantify different types of flow motion with regard to dispersive mixing. While both approaches offer insights into dispersive mixing, practical applicability in optimizing flow dynamics for real mixer geometries under realistic conditions remains limited. Neither method accounts for dissipative heating, nor do they evaluate distributive mixing processes.

In the context of distributive mixing processes, statistical evaluation is a widely used technique for quantifying mixing efficiency. Statistical measurement methods of mixing metrics typically entail computations involving the standard deviation and variance of a massless particle distribution. For this purpose, the trajectories of the massless tracer particles within the prevailing velocity field are calculated using the particle tracking method. The result is the spatial distribution of tracer particles within the mixing element. Various approaches for assessing mixing metrics based on statistical evaluation are documented in the literature, as summarized in [7]. Nevertheless, Phelps and Tucker [8] demonstrated that all statistical measures of mixing quality exhibit numerical limits. This phenomenon stems from the discretization of the flow cross-section into finite surface elements, resulting in limit values contingent upon the quantity of surface elements and total particle numbers. Consequently, statistical measures of mixing metrics often rapidly attain a near-constant value and exhibit negligible further alteration, despite ongoing improvements in mixing efficiency [9].

To address this limitation, Erb [9] proposed a geometric approach as a mixing metric that reconstructs an interface from particle positions obtained via the particle tracking method. The particles represent the flow behaviour of the fluid within the considered domain. The so-called *Delauny* triangulation is used to reconstruct an interface between different particle types representing different components. *Delauny* triangulation is a common method for creating a triangle mesh from a series of points [10]. Notably, this algorithm is devoid of dependence on a finite number of surface elements, thereby obviating numerically determined limit values. The efficiency of this approach was successfully demonstrated with respect to evaluating pressure drop and dissipative heating on a distibutive mixing element. Two operating points were investigated and each of these operating points required a change in the mixer geometry, despite utilizing the same material. The optimization was carried out using the design of experiments method (DoE), i.e., an optimization procedure based on phenomenological and non-physical correlations is presented. The method becomes inaccurate in the prediction when the number of metrics with unknown correlation relations increases. This means the correlations are not generally valid. Additionally, changes in geometry can result in different flow behavior, which cannot be accurately captured through DoE. This calls into question the predictive accuracy of such correlations. With the ever-increasing computing performance, optimization should be performed by using CFD simulation.

However, CFD simulations often cannot be automated when it comes to generating complex, three-dimensional flow domains. As in [9], current simulations require an extensive pre-processing step due to the existing interface between CAD (computer-aided design) software and the CFD simulation software. A complex geometry of an element is often designed in CAD software, which than is used to create a volume mesh that discretize the fluid domain for the simulation. If changes in the geometry are required, the geometry must be redesigned by the user in the CAD software and a new volume mesh must be created in the pre-processing step. In [11], a method is introduced that minimises the effort of the pre-processing step in CFD. The shape of the mixing element can be changed without having to redesign the CAD geometry for a new iteration loop. In other words, the volume mesh of the fluid domain is parameterized and updated, not the CAD geometry. This method can be used for automated optimization, as the user does not have to redesign the CAD geometry by himself. However, the method can only be applied to cuboidal calculation areas, which significantly limits its application to other, more complex geometries, as mixing elements.

In the state of the art, there are virtually no studies on the holistic consideration of the mixing process in the SSE. Nevertheless, it is imperative to consider both processes (dispersive and distributive) to ensure the good quality of extrudates. This is due to the inherent interplay between effective dispersive mixing and suboptimal distributive mixing. This competition shows that there is no single optimal solution, but that a compromise is necessary, which requires extensive and time-consuming research. To confront this problem, a genetic algorithm for an automatic optimization of a dynamic mixing element is used in [12]. Instead of a parameterised volume mesh as in [11], the CAD of a dispersive mixing element is parameterised, to consider a three-dimensional complex geometry. The optimization is handled by *Ansys Fluent*. For the evaluation of the flow results, several metrics are introduced to perform a good distributive and dispersive mixing process. In terms of the material distribution, a scalar is introduced as a transport parameter, which has the value 0 or 1 at the inlet of the mixing element. With an given velocity field, the scalar exhibits values between 0 and 1, with 0.5 indicating a perfectly distributive mixture. This is an abstract approach and hardly takes into account the spatial distribution of the material. Nonetheless, the results show the feasibility of automatic optimization based on dispersive and distributive mixing processes. However, the study [12] only focuses on a dispersive mixing element, but the distributive mixing part is negligible in a dispersive mixing element. Rather, the distributive mixing process should be optimized as a function of the dispersive mixing process on a distributive mixing element. In addition, the dispersive mixing geometry and its chosen geometry parameter in [12] induce hardly any relevant elongational flows, which limits the mixing effect on highly viscous materials.

The aim of this work is to develop an universally automated optimizing tool for mixing elements based on CFD simulation to improve distributive and dispersive mixing performance within a realistic time frame. To achieve this, an automatic workflow is created between several software programs. This means that mixing elements are parameterized and interfaces between the different software are bridged. A genetic algorithm is used for the optimization, which integrates a comprehensive evaluation of the mixing process, including appropriate quantities of interest as mixing metrics. The reconstruction algorithm described in [9] is used to quantify the distributive and the kinematic evaluation based on [5] is used to quantify the dispersive mixing process. These mixing metrics need to be maximized, indicating a high mixing effect. Both flow domains of the mixing elements are simulated and connected in series, where a distributive mixing element is placed behind a dispersive element. This means that the distibutive mixing element is designed depending on the conditions created by the dispersive mixing element. The process conditions such as the pressure drop and temperature gradients are also taken into account as mixing metrics, which have to be minimized for an economical extrusion process. The optimization identifies a set of geometry parameters, a circumferential velocity of the screw, and a process temperature that provides the best compromise for achieving effective mixing for the given material system. This holistic, automated optimization approach offers a prediction of generated mixing elements while eliminating the time- and cost-intensive pre- and post-processing in simulations and trial-and-error experiments.

## 2. Materials and Methods

**Figure 1:**
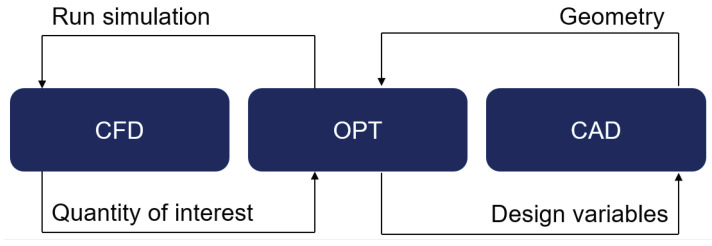
The optimiser (OPT) retrieves a geometry described by design variables (DVs) from the CAD (computer-aided design) software and starts a CFD (computational fluid dynamics) simulation. The results are the quantities of interest that OPT uses to select new DVs and retrieve a new geometry.

The developed optimization process of the automated optimizer for mixing elements in this work is split into three steps and is shown in Figure 1. The optimiser (OPT) starts the process and manages the interface between the different software. It assigns values to the set of geometry parameters called design variables (DVs). A geometry, parameterised with the DVs, is stored in computer-aided design (CAD) software. The geometry is changed according to the DVs. The OPT then starts a computational fluid dynamics (CFD) simulation with the new geometry and computes the mixing metrics, the so-called quantities of interest (QOI), in a post-processing step. In respect to the result, the OPT changes the DVs accordingly, to maximize (or minimise) the QOI. A new loop starts until the optimum is found. The procedure is described in more detail in the following.

### 2.1. OPT—Multi-Objective Optimization

Generally, an optimization problem minimizes or maximizes a function object $f(\vec{x})$ as follows:

(1) $$\mathrm{find}\;\vec{x}=\left[\begin{array}{c} x_{1}\\ x_{2}\\ \vdots \\ x_{n} \end{array}\right]\;\mathrm{which}\;\;\left.\begin{array}{c} \min\\ \max \end{array}\right\}\;f_{k}\left(\vec{x}\right)\;\mathrm{with}\;k=1,2,...,q$$

while satisfying the constraints ${\vec{x}}_{l}\leq \vec{x}\leq {\vec{x}}_{u}$, where vector terms are marked with an arrow. The DVs are defined as a *n*-dimensional vector $\vec{x}\in R$. The constrains are specified with the *n*-dimensional vector ${\vec{x}}_{u}$ as the upper and ${\vec{x}}_{l}$ as the lower bound. These bounds define the sets of allowed values for the elements of $\vec{x}$ and is called the design space. A specific set of values within the design space is known as the design point (DP) [13].

Various approaches exist for addressing the optimization problem represented by Equation (1). Each of these methods involves iterating over the variables in $\vec{x}$. Essentially, an initial DP is assigned to the parameter within $\vec{x}$, followed by the calculation of the QOI. Subsequently, an algorithm is employed to produce a new set of $\vec{x}$ values, aiming to decrease or increase the QOI. The optimizing methods to be chosen depends on the problem. In this work, several QOI (q>1 in Equation (1)) are presented as distributive and dispersive mixing metrics as well as pressure drop and temperature gradients. This means a multi-objective optimization problem is present with competing objectives (high dispersive mixing leads to undesired high temperature gradients).

The competing behaviour of a multi-objective problem does not lead to a singular optimal solution, but comprises a series of solutions (or DP) known as a Pareto front. With a feasible set of criterion vectors $\vec{Y}$, such that

(2) $$\vec{Y}=\vec{y}\in R^{m}:\vec{y}=f\left(\vec{x}\right)$$

and the definition of a point $y^{\prime \prime }\in R^{m}$ which is dominate to another point $y^{\prime }\in R^{m}$, written as $y^{\prime \prime }≻y^{\prime }$, the Pareto front can then be written as

(3) $$P\left(\vec{Y}\right)=y^{\prime }\in \vec{Y}:y^{\prime \prime }\in \vec{Y}:y^{\prime \prime }≻y^{\prime }\neq y^{\prime \prime }=0$$

The points that form the Pareto front are classed as non-dominated [13].

**Figure 2:**
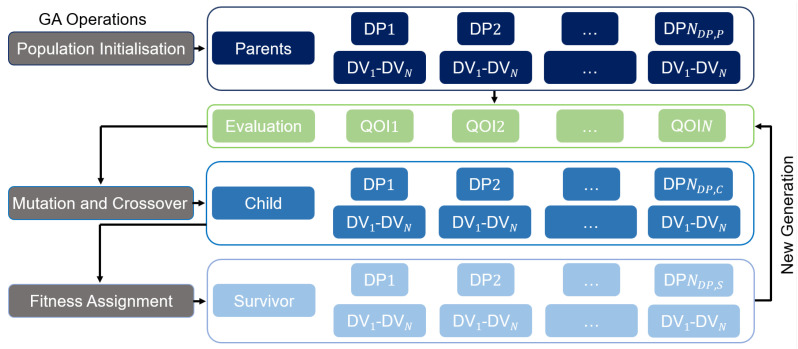
The procedure of the GA algorithm. It starts with a population (parents) consisting of $N_{DP,P}$ design points (DPs), each with *N* design variables (DVs). These are evaluated through $N_{DP,P}$ simulations. GA operations then generate a number of $N_{DP,C}$ new DPs, where $N_{DP,P}\neq N_{DP,C}$ may apply. Fitness is assessed, survivors are selected ($N_{DP,S}$), and a new generation is evaluated.

To optimise a problem with several QOI, a genetic algorithm (GA), in particular the moga (multi-objective genetic algorithm) method, is used in this work. The GA is a non gradient-based algorithm and based on Darwin’s theory of survival of the fittest [13]. The algorithm predicts the evolutionary process by applying mathematical analogies and is shown schematically in Figure 2. That is, the algorithm starts with a number of population $N_{DP,P}$ (one population represents one DP), which represents the first generation (parents) and evaluates the population (calculates the QOI). It then performs a crossover and mutation from the different populations, creating $N_{DP,C}$ new DVs (children), where $N_{DP,P}\neq N_{DP,C}$ can apply. The crossover process can be interpreted as a combination of the parents’ values. The mutation randomly selects a set of DVs within a population and changes the values. This new child population is evaluated by assessing the fitness (higher fitness is better) of each new DP and replacing the better DVs compared to the first population resulting in $N_{DP,S}$ DP. This represents the next generation of a population. The loop starts again and continues the process of crossover and mutation on the next generation until it converges or the selected stop criterion is reached. Finally, the algorithm identifies a set of DPs that minimize/maximize the QOI of the optimization problem. The moga method is particularly well suited for multi-objective problems, as it is specifically designed to avoid problems with aggregating and scaling objective function values and converting them into a single objective [13]. Please refer to [14,15] for a detailed discussion of the GA.

In this study, *Dakota* version 6.13 serves as optimization software [13]. It is a multilevel parallel object-oriented framework for design optimization, parameter estimation, uncertainty quantification, and sensitivity analysis. *Dakota* is available as open-source software under the GNU Lesser General Public License. It has been widely used in various fields, including climate modeling, computational materials science, energy systems, and others. *Dakota* provides built-in functionality for GA, making it particularly well-suited for the high-dimensional optimization problem addressed in this work.

Several QOI are considered in this work: the dispersive and distributive mixing metrics, which need to be maximized whilst maximizing the temperature gradient and the pressure drop. The QOI are described in more detail in Section 2.3. The DVs are the circumferential velocity of the screw as well as a set of geometry parameters of the mixing elements, which are described in more detail in Section 2.2.

### 2.2. CAD—Parameterization of the Mixing Elements

**Figure 3:**
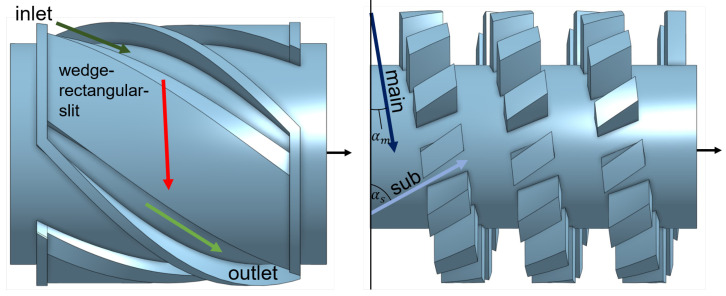
Left: Dispersive mixing element consisting of a total of three inlet (dark green) and outlet (light green) channels. The red arrow marks the wedge-rectangular slit. Right: Saxton element as the distributive mixing element, with one main channel with a helix angle $\alpha_{m}$ to convey and six side channels with $\alpha_{s}$ to separate the melt flow. The black arrows indicate the direction of extrusion.

Two common mixing element are investigated in this work and shown in Figure 3. On the left sidem a dispersive mixing element is depicted, consisting of an inlet and outlet channel which are marked as blue arrows. Between them is a wedge that merges into a rectangular slit (red arrow). Without the wedge, no relevant elongation deformation would be present within the rectangular slit. Due to the wedge, which causes a cross-section reduction, a superposition of shear and elongational deformations are induced on the melt. The element in Figure 3, left, has in total three inlet and outlet channels.

The Saxton element as a distributive mixing element is illustrated in Figure 3, right. It is designed in such a way that the flow is separated into a main (dark blue arrow) and side channel (light blue arrow). There, the main melt-conveying channel is indicated by the helix angle $\alpha_{m}$, and the side channel distributes the melt flow ($\alpha_{S}$). The element shown in Figure 3, right, has one main and seven side channels. The black arrow shows the extrusion direction.

To automatize the optimization mixing process, parameterized CAD geometry of the investigated mixing element is required. The challenge is to describe a three-dimensional complex geometry, as shown in Figure 3, with suitable geometry parameters. The parameters must change the geometry in such a way that they result in a controlled influence on the melt flow. Due to the complexity of the geometries, certain assumptions must be made and some dependencies must be set in order to be able to describe the geometries meaningfully with a few set of parameters.

**Figure 4:**
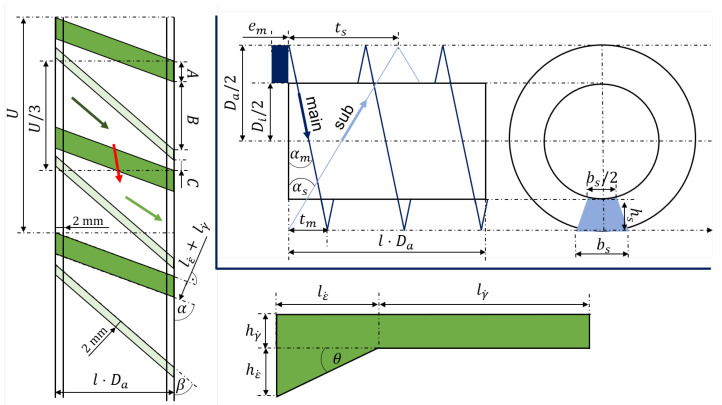
Left: Parameterized unwinding of the dispersive mixing element and the wedge-rectangular slit (red arrow, bottom right) with three inlet and outlet channels (green arrows) and the wedge height $h_{\dot{\varepsilon }}$, the wedge angle θ, and the wedge length $l_{\dot{\varepsilon }}$. The rectangular slit has a length of $l_{\dot{\gamma }}$ and a height of $h_{\dot{\gamma }}$. The element has a circumference *U*. The length is determined by the barrel diameter $D_{a}$ with $l\;\cdot \;D_{a}$, l∈N. With $A=(l_{\dot{\varepsilon }}+l_{\dot{\gamma }})\;\cdot \;\cos\alpha$ follows $b,c=s_{f}\;\cdot \;\left(U/3-A\right)$ with a scaling factor $s_{f}=0.8,0.15$. The light-green areas are barriers and α=32.14°. Top right: The parameterized Saxton element has a length *l* with a screw $D_{i}$ and a barrel $D_{a}$ diameter. The side channel (light blue) is described by the perpendicular flight width $b_{s}$ and height $h_{s}$, which is surrounded by a helix defined by angle $\alpha_{s}$ and pitch $t_{s}$. The main-channel results of the flight (dark blue) described by the flight width $e_{m}$ and height $h_{m}$ which is surrounded by a helix defined by angle $\alpha_{m}$ and pitch $t_{m}$.

In [5], the main influencing parameters are introduced of the dispersive mixing element, derived on a two-dimensional wedge-rectangular slit system, as shown in Figure 4 in green, bottom right. The parameters are the angle of the wedge θ, the length $l_{\dot{\gamma }}$, and the height $h_{\dot{\gamma }}$ of the rectangular slit. In order to use these parameters as the DVs for optimizing a 3D geometry, a so-called unwinding must be carried out, which can be seen in Figure 4, left.

The dark-green areas represent the wedge-rectangular slit system, and the light-green areas are barriers (with a fixed width of 2 mm) to force the melt in the right direction across the slit. The orientation of the slit is determined by the angle α. This angle is kept constant with α=32.14°, which results in three inlet and outlet channels. This design has been proven to be well-suited in [16]. Based on the circumference *U* defined by a barrel diameter $D_{a}$, the unwind can be divided into U/3 sections, where U/3=A+B+C. The parameter *A* depends on length of the wedge $l_{\dot{\varepsilon }}$ and the length of the rectangular slit $l_{\dot{\gamma }}$ that

(4) $$A=(l_{\dot{\varepsilon }}+l_{\dot{\gamma }})\;\cdot \;\cos\alpha .$$

Depending on *A*, the length of B=0.8·(U/3−A) and C=0.15·(U/3−A) are determined, each with a scaling factor to maintain the ratios between A,B,C when *A* changes. The orientation of the barriers β results from A,B,C. The parametrization of the wedge-rectangular slit can be found in more detail in Figure 4, bottom right. It is constructed in such a way that the height $h_{\dot{\varepsilon }}$ of the wedge results for a given θ and $l_{\dot{\varepsilon }}$. To change $h_{\dot{\gamma }}$, the screw diameter of the element varies ($D_{i}=f\left(h_{\dot{\gamma }}\right)$). As the barrel diameter is a machine-dependent variable; it is kept constant. The length of the element is not considered as a DV. The QOI used to quantify the dispersive mixing process (see Section 2.3.3) are not greatly affected by the length of the element. Rather, the length $l_{\dot{\gamma }}$ and height $h_{\dot{\gamma }}$ of the rectangular slit are mainly responsible for high deformations.

The most important parameters to describe the geometry of a Saxton element are introduced in [9]. These are the helix angles of the two channels, defined by $\alpha_{m}$ for the main and $\alpha_{s}$ for the side channel. These parameters are defined as DV for the Saxton element, as well as the perpendicular flight width $b_{s}$. The design of the Saxton geometry is based on a screw thread. To construct a Saxton element, it must be divided into several threads defined by helices. The characteristic parameters of the Saxton are illustrated in Figure 4 in blue, top right. The side view is shown on the left and the front view on the right side. The extrusion direction is from left to right. In case of the main channel, the flight, with a width of $e_{m}$ and a height of $h_{m}$ (area marked in dark blue) is first formed by a helix. The helix is described by the helix angle $\alpha_{m}$ and the pitch $t_{m}$. The flight width remains constant with $e_{m}=8mm$ and the height results from the screw $D_{i}$ and barrel $D_{a}$ diameter of the element with $h_{m}=0.5\;\cdot \;\left(D_{a}-D_{i}\right)$. The perpendicular flight width $b_{m}$ results from $t_{m}$ where $t_{m}=\pi \;\cdot \;D_{a}\;\cdot \;\tan\alpha_{m}$. The ribs of the Saxton element are constructed by the side channel. For this, the side channels (light blue) with its perpendicular flight width $b_{s}$ and its height $h_{s}=h_{m}$ are cut out from the previously imprinted main flight. Therefore, the flight width $e_{s}$ results not only from $b_{s}$ but also from $\alpha_{m}$ and $\alpha_{s}$. To reduce the degrees of freedom, the number of main channels $N_{m}=1$ and side channels $N_{s}=6$ remain constant, which has proven a successful mixing element in [9]. The barrel diameter and the screw diameter are kept constant as well in this case, since these parameters depend on the machine itself. In addition, both elements are designed in such a way that the radial screw clearance is neglected. An overview of the characteristic geometry parameters and their dependencies on the DVs for the Saxton and the dispersive mixing element are listed in Table 1.

In this work, the CAD software *onshape* version 1.193 is used to design the mixing elements. *Onshape* is a professional, cloud-native CAD platform that can be accessed free of charge with the education license. Users are able to interact with the system via a web browser. *Onshape* offers a robust integration framework for connecting systems to *onshape* via secure REST APIs. It is independent of an operating system and facilitates the automation of processes. In this work, *python* version 3.10 is used as script language with the *onshape*-client package. The script can configure the CAD geometry and extract it into a suitable file format, such as *stl*. The *stl* files are used to define the fluid domain for the CFD simulation, which is described in the next section, Section 2.3.

However, meshes generated directly from CAD-exported *stl*-files are often inadequate for meshing programs, typically requiring further processing to ensure a high-quality volume mesh. This additional step is normally completed in a separate software. It involves generating a surface mesh of uniform triangles, which enhances the stability and quality of the resulting volume mesh. To streamline this process, a pattern is applied to the geometry surface within the CAD software itself. This means that an area of 0.104 m$m^{2}$ is simply cut out of the surface of the screw all the way around using a helix. While this pattern does not alter the geometry, it affects the *stl* export by orienting the triangles uniformly. As a result, almost equilateral triangular elements can be generated directly from the CAD software, ensuring stable and reliable meshing across varying geometries without the need for additional software.

### 2.3. CFD—Quantification of the Mixing Process

To predict the flow behavior of a plastic melt in the extrusion process, the transport parameters’ mass $\dot{m}$, velocity $\vec{u}$ and temperature *T* of a fluid are balanced on a control volume $V_{k}$ so that a general transport equation for a stationary flow is as follows:

(5) $$\nabla \;\cdot \;\left(\rho \vec{u}\phi \right)=\nabla \;\cdot \;\left(\Gamma \nabla \phi \right)+q_{\phi }$$

where the left-hand side describes the convection and the right-hand side the diffusion transport [17]. Here, ϕ is the respective transport parameter, Γ is a proportionality coefficient and $q_{\phi }$ is a source term. The fluid domain consists of k=1,2,…,n volume elements, which results in a linear system of *n* equations after the discretization of the equations.

If ϕ=1,Γ=0 and $q_{\phi }=0$, Equation (5) becomes the continuity equation

(6) $$\nabla \vec{u}=0$$

valid for incompressible fluids, which is assumed for a plastic melt flow [18]. If $\phi =\vec{u},\Gamma =\eta$ and $q_{\phi }=-\Delta p+\vec{g}$, the momentum equation results from (5) to

(7) Δp=∇τ

where

(8) $$\tau =\eta \nabla \vec{u}$$

with the pressure drop Δp and the dynamic viscosity η as a material parameter. Gravitation $\vec{g}$ and inertia $\nabla {\vec{u}}^{2}$ can be neglected compared to the high viscose forces (Reynolds number Re<<1) in plastic melt flow. The high viscosity indicates a diffusion-dominated flow, as momentum transport is primarily governed by the diffusive term $\nabla \;\cdot \;(\eta \nabla \vec{u})$, which represents the internal friction within the fluid. In (8), the common linear transport approach of the stress tensor τ for a Newtonian fluid is applied with η=const., where bold letters mark a tensor. However, a plastic melt is a shear-thinning fluid and the shear-stress tensor is non-linear to the velocity gradient tensor L [19]. For this, L is introduced as

(9) $$L=\nabla \vec{u}$$

and can be split into a symmetric (D) and anti-symmetric (W) tensor

(10) L=D+W

where

(11) $$D=\frac{1}{2}\left(L+L^{T}\right).$$

According to the index notation, (11) gives

(12) $$D_{ij}=\frac{1}{2}\left(\frac{\partial u_{i}}{\partial x_{j}}+\frac{\partial u_{j}}{\partial x_{i}}\right)$$

for i,j=1,2,3, which can be also expressed by

(13) $${\dot{\gamma }}_{ij}=2D_{ij}$$

with the deformation gradient tensor ${\dot{\gamma }}_{ij}$, which is an alternative formulation of $D_{ij}$. To describe the behavior of a shear-thinning fluid, the viscosity can be express as a function of the magnitude of ${\dot{\gamma }}_{ij}$ and (8) changed to a generalized transport model

(14) $$\tau_{ij}=\eta (|\dot{\gamma }\left|\right){\dot{\gamma }}_{ij}$$

where

(15) $$|\dot{\gamma }|=\sqrt{\sum_{i}^{3}\sum_{j}^{3}{\left(\frac{\partial u_{i}}{\partial x_{j}}+\frac{\partial u_{j}}{\partial x_{i}}\right)}^{2}}.$$

Nonetheless, Equation (14) can only describe the non-Newtonian (shear-thinning) viscosity, while normal stress effects and time-dependent effects (elastic effects) are not taken into account [19]. This means that the plastic melt is treated as a viscous material. However, it is common practice to simplify the flow behavior of plastic melts in this way and it is useful for practical applications such as process prediction [18].

To solve (14), an approach to determine the viscosity as a function of $\dot{\gamma }$ is necessary. In this work, the Carreau–Arrhenius approach

(16) $$\eta (|\dot{\gamma }\left|\right)=\frac{a\;\cdot \;\alpha_{T}}{(1+b\;\cdot \;\alpha_{T}\;\cdot \;|\dot{\gamma }{\left|\right)}^{c}}$$

is used, with the temperature-dependent Arrhenius shift factor

(17) $$\alpha_{T}=\exp\left(\frac{E}{R}\left(\frac{1}{T}-\frac{1}{T_{0}}\right)\right)$$

to include the temperature dependence at the viscosity. In Equation (16), a,b and *c* are model parameters and must be approximated for each material at a reference temperature $T_{0}$. Finally, this also results in the activation energy *E* in (17). The universal gas constant is denoted as *R*. Here, *a* characterizes the viscosity level (zero-shear viscosity), b quantifies the elastic properties (relaxation time) and c shows the extent of the shear-thinning flow behavior of the melt. Further possible shear-thinning approaches are discussed in [20].

In a non-isothermal simulation, the energy transport equation must be also taken into account, which is derived from (5) with $\phi =T,\Gamma =\lambda /c_{p}$ and $q_{\phi }=0$ to

(18) $$\nabla \;\cdot \;\left(\vec{u}\;\cdot \;T\right)=\nabla \;\cdot \;\left(\frac{\lambda }{c_{p}\rho_{F}}\nabla T\right)$$

with the fluid density $\rho_{F}$, the heat conductivity λ and the heat capacity $c_{p}$ as constant material parameters. From Equation (18), it can be seen that temperature is transported through the flow by convection, represented by the term $\nabla \;\cdot \;(\vec{u}T)$. Heat diffusion occurs through the term $\nabla \;\cdot \;\left(\frac{\lambda }{c_{p}\rho_{F}}\nabla T\right),$ which describes the conductive heat transfer within the fluid.

The system of equation (SOE) consisting of (6), (14) and (18) needs to be discretized and solved numerically with a suitable set of boundary conditions (BCs). For this purpose, the finite volume method is used, which discretizes the equations and generates a linear equations of systems to solve the flow parameters $p,\vec{u}$ and *T*.

In this work, this is performed by the software *openFoam*^®^version v2212. It is produced by OpenCFD Ltd, Berkshire, UK, which is freely available, licensed under the GNU General Public Licence. The software provides a highly flexible and extensible framework for CFD, in which it is possible to implement user-specific models, as a non-Newtonian viscosity approach. To predict the flow behavior, a pressure-based solver is used valid for steady-state simulations of incompressible flows in a single rotating frame (SRF) [21]. During the simulation, the flow parameters are calculated separately. The parameters are therefore calculated iteratively, whereby the conservation of momentum and mass is guaranteed [22].

Using a steady-state solver is a common procedure for process prediction in polymer processing. It provides reliable insights into the process and keeps computational costs low compared to transient and/or dynamic mesh solvers. However, this solver is valid for non-isothermal simulations. For this purpose, the solver is extended within this work by Equation (18) as an extra transport equation. In addition, Equation (16) is implemented as a new viscosity model within the *openFoam* ^®^architecture.

#### 2.3.1. Setting up the Simulation Cases

**Figure 5:**
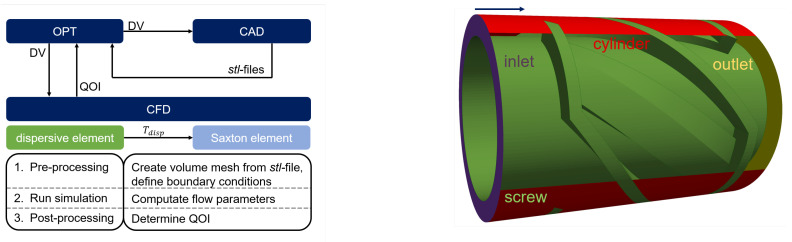
Left: An overview of the optimization tool and CFD simulation process. The dispersive mixing element simulation, divided into three steps, is performed first, followed by the Saxton element simulation using the initial $T_{\mathrm{disp}}$ profile from the dispersive simulation. Right: Colored patches of the dispersive mixing element. The fluid (plastic melt) flows through the inlet and exits at the outlet. The cylinder and screw are both the *wall* type. A stationary cylinder is considered in the reference frame and $\vec{u}=0$ is applied on the screw. The blue arrow marks the flow direction.

The CFD simulation as part of the automated optimization process consist of two simulation cases: the dispersive mixing and Saxton element case. The simulations steps of both cases are equal and listed in Figure 5, left. In a first step, a volume mesh of the mixing element is created which discretize the fluid domain in $V_{k}$ volume elements. The domain are constructed by the *stl* files which are extracted from the respective CAD geometry with the *onshape* API-client. These files serve as input for *cfMesh*^®^version 1.1.1, which generate the volume mesh. *cfMesh*^®^is a cross-platform library for automatic mesh generation that is built on top of *openFoam*^®^and licensed under GPL [23].

**Figure 6:**
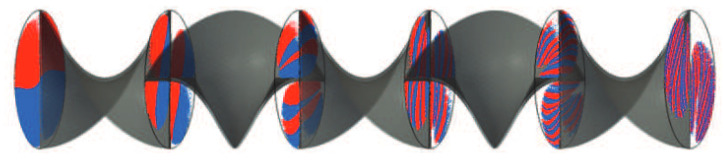
Two particle clouds (red and blue) within a fluid domain exemplary of a kenics mixing element illustrate the interface between the clouds at the inlet ($\lambda_{0}$) and outlet ($\lambda_{1}$), where $\lambda_{0}<\lambda_{1}$ applies. The flow direction is from left to right [9].

To solve the SOE, BCs need to be specified. For this, so-called *patches* are defined within the volume mesh, listed in Table 2 and illustrated in Figure 5, right, exemplifying the dispersive mixing element. Both geometries consist of the *patch*-type *wall* (screw, cylinder), and both have the outlet and the inlet of the volume mesh defined as *patch*. While the dispersive mixing element has one inlet, the Saxton has two inlet *patches* (left and right) due to the QOI (see Figure 6). The BCs are defined either via the derivation of the flow parameters (Neumann-BC) or with initial values of the parameters (Dirichlet-BC). The initial velocity is defined at the boundaries in accordance with the SRF with an initial inlet velocity value determined by a given mass rate $\dot{m}$, the inlet area *A* of the mixing element, and the flow density $\rho_{F}$.

After the pre-processing step, the simulation runs a second step; the first is for the dispersive mixing and the second for the Saxton element. This is to map the received $T_{disp}$-profile at the outlet *patch* of the dispersive mixing element to the inlet *patches* of the Saxton element as the initial profile.

To ensure stable simulations despite varying geometries and differing volume meshes, a four-step simulation approach with increasing numerical complexity is employed. The first step involves an isothermal simulation using only the Carreau viscosity model (Equation (16) without the Arrhenius shift factor). Once this initial simulation converges, the second step is conducted, a non-isothermal simulation, utilizing the converged flow profiles from the previous step as the initial conditions while still applying the Carreau model. Upon convergence of the second simulation, in the third step, a simulation is performed with the Carreau–Arrhenius model, which introduces greater complexity due to the temperature dependency.

In the final phase, accounting for the temperature-dependent viscosity model can lead to instabilities in the simulation. To address this, an additional parameter, $\alpha_{\eta }$, is introduced to control changes in the viscosity field (similar to the under-relaxation factor of the flow parameters. The viscosity is updated as

(19) $$\eta =\alpha_{\eta }\eta_{t}+\left(1-\alpha_{\eta }\right)\eta_{t-1},$$

where *t* represents the simulation time step. Initially, $\alpha_{\eta }=0.7$ is used; once convergence is achieved, a subsequent simulation with $\alpha_{\eta }=1.0$ is run. This multi-step approach ensures stable and converged flow simulations even with changing geometries.

After the simulation has converged, the final step begins: post-processing. This means that after the simulation run of both mixing elements, the QOI are calculated in order to quantify the mixing effect. One QOI is the pressure drop

(20) $$\Delta p=\sum_{k}\Delta p_{k},$$

which is summed from the two elements with k={dispersive,distributive} and minimized in order to ensure low energy costs. To quantify the dispersive and distributive mixing processes, a set of metrics are considered and described in more detail in the following.

#### 2.3.2. Distributive Mixing Metric

A geometric method is used to quantify the distributive mixing effect. In particular, Erb’s interface reconstruction [9] is applied by using the particle tracking method, calculated in the Lagrangian frame. Thereby, a particle *p* is defined by the position of its center ${\vec{x}}_{p}$ and its velocity ${\vec{u}}_{p}$. In a Lagrangian frame, each particle vector $\vec{x_{p}}$ is calculated from

(21) $$\frac{d{\vec{x}}_{p}}{dt}={\vec{u}}_{p}$$

and the motion of the particles is governed by Newton’s equation

(22) $$m_{p}\frac{d{\vec{x}}_{p}}{dt}=\sum_{i}{\vec{F}}_{i}$$

with the mass of the particle $m_{p}$ and the forces $F_{i}$, which act on the particle. The particle should completely reflect the flow behavior within the considered fluid domain. Therefore, particles with a diameter $d_{p}<100\;\mu m$ are considered. The density of the solid particles is assumed to be equal to that of the fluid density. Additionally, a one-way coupling system is considered and particle–particle interactions are neglected. Because of Re<<1, the buoyancy forces are not taken into account and the particles are not adsorbed on the walls. The dominant force acting on the particle is the drag force ${\vec{F}}_{D}$ from the fluid, which can be expressed as

(23) $${\vec{F}}_{D}=-m_{p}\frac{{\vec{u}}_{p}-\vec{u}}{\tau_{p}}$$

where $\tau_{p}$ is the relation time of the particle. This is the time it takes for a particle to respond to changes in the local flow velocity $\vec{u}$, calculated in the Eulerian frame, discussed in Section 2.3. To use the reconstruction algorithm developed in [9], two particle clouds are considered. An interface $\lambda_{int}$ is reconstructed between the particles of the clouds at the beginning (inlet: $\lambda_{0}$) and at the end (outlet: $\lambda_{1}$) of the fluid domain.

To illustrate this, two particle clouds (colored red and blue for differentiation) are shown in Figure 6 within a fluid domain of a kenics mixing element, a static mixer investigated in [9]. The flow direction is from left to right. At the inlet, the particle clouds are still separated (top and bottom) but are mixed due to the $\vec{u}$-profile within the kenics element. The interface at the inlet and outlet are reconstructed, where $\lambda_{0}<\lambda_{1}$ applies.

The interface are based of the particle positions ${\vec{x}}_{p}$ using *Delauny* triangulation. This means that triangles are created whose vertices represent the particle positions. All triangles whose vertices contain particles from the same cloud are deleted. Finally, the centres of the circumcircles of the triangles are calculated and connected to the centres of their neighbours. This connection only takes place via triangle edges whose vertices consist of particles from different clouds. The connections are summed up and correspond to the reconstructed interface between the particles of the different clouds. If the interfaces of inlet and outlet are set in relation to each other, the result is the reconstructed interface

(24) $$\lambda_{int}=\frac{\lambda_{0}}{\lambda_{1}}$$

as a QOI, which measure the distributive mixing effect. The higher $\lambda_{int}$ becomes, the better the distributive mixing. Further details on the algorithm can be found in [9]. In this work, the application *icoUncoupledKinematicParcelFoam* implemented in *openFoam*^®^which solved Equations (21)–(23) is used to track the particles within a given $\vec{u}$-profile and is referred to in the following as Lagrangian particle simulation, LPT.

The coefficient of variation of the temperature $CoV_{T}$ at the outlet of the Saxton element is calculated as an additional metric for distributive mixing. A lower $CoV_{T}$ corresponds to a lower temperature gradients and therefore a more homogeneous temperature distribution across the outlet.

#### 2.3.3. Dispersive Mixing Metrics

To quantify the dispersive mixing effect, a kinematic evaluation of the $\vec{u}$-profile based on [5] is applied. This means that the two types of flow present in the dispersive mixing element, shear and elongational flows, are considered in detail. Within a shear flow, the secondary diagonal of (12) are considered, where the magnitude of the shear-stress tensor results in

(25) $$|\dot{\gamma }{|}_{\dot{\gamma }}=\sqrt{\sum_{i}^{3}\sum_{j\neq i}^{3}{\left(\frac{\partial u_{i}}{\partial x_{j}}+\frac{\partial u_{j}}{\partial x_{i}}\right)}^{2}}$$

with $\dot{\gamma }=\frac{\partial u_{i}}{\partial x_{j}}$ known as shear rate. The fluid includes an elongational deformation in flow direction which is described with the main diagonal of (12). This means the magnitude gets to

(26) $$|\dot{\gamma }{|}_{\dot{\varepsilon }}=\sqrt{3}\;\cdot \;\frac{\partial u_{i}}{\partial x_{i}}$$

where ${\dot{\varepsilon }}_{i}=\frac{\partial u_{i}}{\partial x_{i}}$ for i=1,2,3 known as the elongational rate. If $x_{1}$ is the flow direction, then ${\dot{\varepsilon }}_{1}$ is the elongational rate and ${\dot{\varepsilon }}_{2}={\dot{\varepsilon }}_{3}=-\frac{1}{2}{\dot{\varepsilon }}_{1}$ due to continuity, if an uni-axial elongational flow is present [18].

In case of characterizing the disperisive mixing effect, both type of flows must be considered. Because of the upper bound of shear flow at high viscosity ratios, the elongational flow has a particular significance in highly viscous and laminar plastic melt flow. This means, averaging Equation (15) over all cell volumes is misleading, if only the shear rate is increasing. For this, both Equations (25) and (26) should be considered separately. To obtain a representative scalar describing the total deformation in the domain, the volume integral is calculated for the elongational flow

(27) $${\dot{\varepsilon }}_{tot,i}=\sum_{k=0}^{n}\frac{|\dot{\gamma }{|}_{\dot{\varepsilon_{i}},k}\;\cdot \;u_{k}V_{k}}{u\;\cdot \;V}$$

where i=1,2,3 for all three spatial directions and *n* is the number of volume cells. The three volume integrals for x,y and *z* are summed up using

(28) $${\dot{\varepsilon }}_{tot}=\sum_{i=x}^{3}{\dot{\varepsilon }}_{tot,i}.$$

The volume integral for the shear flow is calculated as follows:

(29) $${\dot{\gamma }}_{tot}=\sum_{k=0}^{n}\frac{|\dot{\gamma }{|}_{\dot{\gamma },k}\;\cdot \;u_{k}V_{k}}{u\;\cdot \;V}$$

with $V_{k}$ as cell volume for each cell *k* and *V* as the total volume of the mesh. The deformation gradients are used as metrics, whereby it is assumed that high shear and elongational rates promote the splitting of the plastic melt. Thus, maximizing the (28) and (29) enhances the likelihood of droplet breakup.

### 2.4. Materials

The materials under investigation are polystyrene (PS), polypropylene (PP), polylactide (PLA), and high-density polyethylene (HDPE), which are characterized in [16,24]. The Carreau–Arrhenius parameters (a,b,c) and the activation energy *E* for the materials, as well as the thermal conductivity λ and the specific heat capacity $c_{p}$, are listed in Table 3. The zero-shear viscosity (marked with *a*), the relaxation time (quantified with *b*), and the shear-thinning flow behavior of the melt (marked with *c*) are varied. The selected materials were chosen to cover a broad spectrum of rheological properties, including significant variations in viscosity and relaxation time, ensuring a comprehensive representation of flow behaviors and deformation responses. Additionally, these materials are commonly used in industrial processes, further highlighting their practical relevance to the study. Details regarding the manufacturers, brand names, and other specifications of the materials used in this study are provided in Table A1.

## 3. Results and Discussion

The automated optimization tool (see Algorithm A1 for detailed information about the automation script) is used in this work to find the optimal mixing geometry and the process conditions for four plastic materials. The optimal process conditions are defined by the maximization of the total shear ${\dot{\gamma }}_{tot}$, the elonagational rate ${\dot{\epsilon }}_{tot}$, the reconstructed interface $\lambda_{int}$, the minimization of the pressure drop Δp, and the covariance of the temperature $CoV_{T}$.

This chapter is structured as follows: First, the settings and assumptions within the respective software (*onshape*, *dakota*, *openFoam*) are outlined. Next, the results of the moga optimizations of the individual plastic materials are discussed separately. In the final subsection, a comparison of the materials is presented, focusing on viscosity and simulation stability.

### 3.1. Settings for Geometries, Simulations and Moga Algorithm

The mixing geometries of both mixing elements (dispersive and Saxton) as well as the operating points are defined within a design space, which is listed in Table 4 as DV. An SSE with an barrel diameter $D_{a}=35\;mm$ is under investigation, which has an screw diameter of $D_{i}=15\;mm$ for the Saxton element. The diameters must be selected once, right at the beginning. This means that the optimization can be carried out for a specific extrude size. In this work, a first-degree polynomial was fitted to an experimentally determined throughput at different circumferential velocities for an extruder size with $D_{a}=35\;mm$ [9]. OPT uses this function as soon as the operating point is changed, so that the mass rate is adjusted accordingly. The mass rate serves as the input for the initial inlet velocity value (see Table 2). It is assumed that a grooved barrel is used in the feed zone of the SSE, so that the throughput is independent of the back pressure. If the optimum diameters of the barrel or the screw are required, they can easily be changed to a DV and be part of the optimization.

The simulation cases are configured as follows: To create the volume mesh in the pre-processing step, a maximum cell size of 0.299 mm is used for both elements with an additional object (hollow-cone) refinement level of 1 for each element. The dispersive element passes through an additional object refinement with a cell size of 0.09 mm in the area of the thin rectangular slit. This provides good stability for meshing and simulation. For the linear solvers, the generalised geometric–algebraic multi-grid (GAMG) algorithm with a Gauss–Seidel smoother is used for the pressure correction equation, a symmetric Gauss–Seidel smoother solves the velocity equation and a stabilized version of the Bi-Conjugate Gradient algorithm (BiCGStab) using a diagonal incomplete Cholesky (DILU) preconditioner solves the temperature equation. To improve the stability of the computation, the under-relaxation factors $\alpha_{i}$ for each parameter *i* are set to $\alpha_{p}=\alpha_{T}=0.3$ for the pressure and temperature, with $\alpha_{u}=0.7$ for the velocity.

The simulation time step of the particle tracking method is Δt=0.1 ms. The two particle clouds are defined as follows: Every second, $10^{6}$ parcels are injected into the fluid domain of the Saxton element in each inlet for a duration of 5 ms. One parcel consists of 1 particle. This means that there are a total of 10,000 parcels in the fluid domain, which is equivalent to 10,000 particles. The particle diameter is set to 100 μm for the inlet *inletLeft* and 100.001 μm for *inletRight*. This is to differentiate between the two particle clouds for the post-processing step. As soon as 5000 particles reach the outlet, the solver stops and the interface between the two particle clouds at the time step 5 ms and the latest time step are determined and set in relation to each other. The higher the number of particles selected, the longer it takes to calculate the interface. A compromise must be found between calculation efficiency and accuracy. However, an end time was specified based on the length of the Saxton element, with values of 8, 16, and 24 corresponding to l=1, l=2, and l=3, respectively.

The OPT settings are as follows: The size of the initial population is set to 15 and the values are created randomly while ensuring uniqueness (*unique_random*). The DVs for the crossover are randomly selected from a specified number of parents, with each offspring receiving shuffled combinations of parent values. For this study, the number of parents is set to 3, the number of offspring to 2, and the probability of a crossover event to 0.8 (*shuffle_random*). The mutations introduce random variation by reassigning a randomly chosen DV from a randomly selected design to a new valid value, independent of its current value. The mutation rate, specifying the probability of a mutation, is set to 0.2 (*replace_uniform*). The fitness is determined by ranking designs based on the number of others that dominate them, with higher fitness corresponding to fewer dominating designs (*domination_count*). To retain designs, the selection process keeps only those with a negated fitness below a limit of 1, with a shrinkage fraction of 0.8 controlling the percentage of retained designs (*below_limit*). As converge method, the *metric_tracker* is used. It tracks various changes in the non-dominated frontier (Pareto front) from generation to generation. When the changes that occur over five generations fall below 0.05% deviation or when a maximum of 1000 iterations is reached, the algorithm stops. The configuration is primarily based on the default settings provided in the *Dakota* user manual [12], which guarantee a robust and efficient optimization process.

Table 4 lists the DV with their constraints and the QOI. Circumferential velocity and temperature are chosen as DVs since they are directly adjustable on the SSE machine, allowing optimization to identify efficient operating settings within realistic constraints, ensuring practical and feasible results.

As discussed in Section 2.1, there is no single optimal solution. Instead, multiple so-called evaluations are performed, with each evaluation resulting in a unique DP consisting of the DV and the corresponding QOI. This means that one evaluation of the moga optimization for a single material generates eight DVs and five QOI. The total number of evaluations *n* depends on the convergence criterion of the moga algorithm. To enable a comparison, the DVs and QOI are normalized using:

(30) $$P_{norm,i}=\frac{P_{i}-P_{min,i}}{P_{max,i}-P_{min,i}}$$

where P= QOI, DV and i=0,1,…,n.

The results are visualized using parallel coordinate plots, a method for representing multi-dimensional data, where each variable (DV and QOI) is depicted as a vertical axis, and the data points are connected by lines. This means that each line represents one moga evolution, i.e., one design point (DP) and the corresponding QOI. This approach effectively captures correlations, trends, and trade-offs. For clarity, the DV and QOI are analyzed separately for the dispersive and Saxton elements. QOI values that were set to zero due to error messages during the moga optimization were excluded from further analysis.

### 3.2. Moga Optimization for HDPE

**Figure 7:**
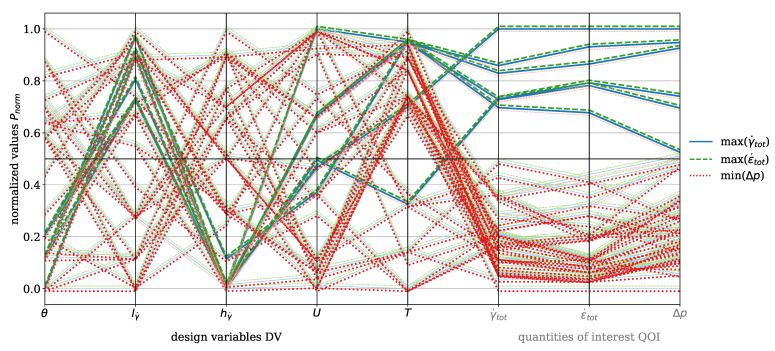
The evaluations of the moga optimization for the material HDPE and the dispersive mixing element using the DV wedge angle θ, rectangular slit length $l_{\dot{\gamma }}$ and height $h_{\dot{\gamma }}$, circumferential velocity *U*, and temperature *T*, and the QOI total shear rate ${\dot{\gamma }}_{\mathrm{tot}}$ and total elongation rate ${\dot{\epsilon }}_{\mathrm{tot}}$ to be maximized, and pressure drop Δp to be minimized, with darker tones indicating values above 0.5 for maximization and below 0.5 for minimization.

In Figure 7, the parallel coordinate plot for the dispersive mixing element is presented for the material HDPE. The DVs include wedge angle θ, rectangular slit length $l_{\dot{\gamma }}$ and height $h_{\dot{\gamma }}$, circumferential velocity of the screw *U*, and temperature *T*. The QOI are total shear rate ${\dot{\gamma }}_{\mathrm{tot}}$ and total elongation rate ${\dot{\epsilon }}_{\mathrm{tot}}$, which are to be maximized, and pressure drop Δp, which is to be minimized. The number of analyzed evaluations is n=45. Darker tones indicate values greater than or less than 0.5, depending on whether maximization or minimization is targeted. For instance, pressure drop must be minimized, which is why values below 0.5 are represented with a dark red, while the total shear rate must be maximized, and values above 0.5 are depicted with a dark blue. A black horizontal line indicates the 0.5 threshold. The blue solid line represents the total shear rate, the green dashed line indicates the total elongation rate, and the red dotted line corresponds to the pressure drop. An offset of +0.001 was applied to the total elongation rate and −0.001 to the pressure drop for improved visualization, as the lines would otherwise overlap directly. This representation is consistently applied in all subsequent plots.

The plot highlights the inherent trade-off between deformation rates (${\dot{\epsilon }}_{\mathrm{tot}},{\dot{\gamma }}_{\mathrm{tot}}$) and pressure drop. High deformation rates are associated with increased pressure drop, while low deformation rates result in reduced pressure drop. This trade-off necessitates a compromise, as clearly illustrated in the plot by observing the dark-blue solid and the dark-green dashed lines corresponding to high deformation rates. These high rates are achieved with a rectangular slit height greater than 0.5 and a rectangular slit length greater than 0.5, which reduce the cross-sectional area through which the polymer melt flows. Consequently, the pressure drop must increase under constant mass-flow conditions. The range of wedge angles can be further specified, indicating that it should be less than 0.2 in order to be associated with higher elongation rates. The plot also shows that no evaluation exists with values exceeding 0.5 for total shear rate and total elongation rate, while maintaining a pressure drop below 0.5. When decreasing the threshold to 0.4, one evaluation line is found. While certain design modifications to the mixing element could reduce pressure drop even more, these would inherently decrease deformation rates and, consequently, the mixing efficiency.

**Figure 8:**
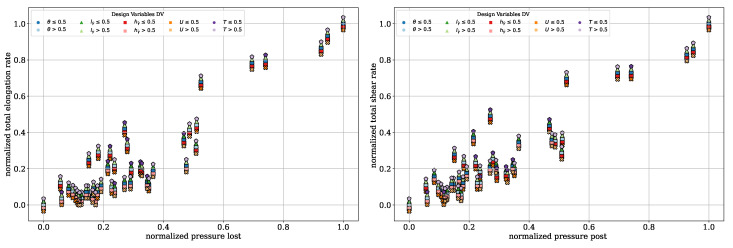
Pareto front for the material HDPE and the dispersive mixing element for the total elongation rate (**left**) and the total shear rate (**right**) versus the pressure drop (normalized). The wedge angle θ, rectangular slit length $l_{\dot{\gamma }}$ and height $h_{\dot{\gamma }}$, circumferential velocity *U*, and temperature *T* are the DVs. The marker shapes represents one DV. Dark tones indicate DV values less than 0.5, while light tones represent values greater than 0.5.

To further illustrate the trade-offs between the QOI, Pareto front diagrams are presented for the dispersive mixing element. These plots highlight the inherent contradictions among the QOI by comparing them directly. In Figure 8, the normalized deformation rates are plotted against the normalized pressure drop. A color distinction is applied based on the DV, including the wedge angle θ, rectangular slit length $l_{\dot{\gamma }}$ and height $h_{\dot{\gamma }}$, circumferential velocity *U*, and temperature *T*. Darker tones indicate DV values below 0.5, while lighter tones correspond to values above 0.5. Each marker shape represents one DV, allowing for the clear identification of the individual DVs. To improve clarity and avoid overlapping points, an offset of Δ=±0.001 was applied to the pressure drop and total elongation rate values.

In Figure 8, left, the dependency between the total elongation rate and pressure drop can be seen. Lower pressure drops correspond to lower elongation rates, and vice versa. The color coding highlights that, particularly for rectangular slit height, the darker red points are located on the right, corresponding to higher pressure drops. Similarly, wedge angles smaller than 0.5 is associated with higher elongation rates. Temperature and circumferential velocity, however, appear to have minimal influence on the QOI within the investigated design space. This indicates that the geometry exerts a dominant influence on the flow behavior. A compromise can be identified with a pressure drop between 0.4 and 0.6 and a total elongation rate between 0.6 and 0.8, which corresponds to three evaluations.

In Figure 8, right, the normalized total shear rate is plotted against the normalized pressure drop. The same dependency observed for the elongation rate is evident here: lower shear rates correspond to lower pressure drops and vice versa. Additionally, $h_{\dot{\gamma }}\leq 0.5$ and $l_{\dot{\gamma }}\leq 0.5$ lead to higher shear rates, while no significant influence from temperature or circumferential velocity is observed. In the range of 0.6 to 0.8 for the total shear rates and 0.4 to 0.6 for the pressure drop, the DVs provide a suitable compromise between achieving high shear rates and maintaining low pressure drop.

**Figure 9:**
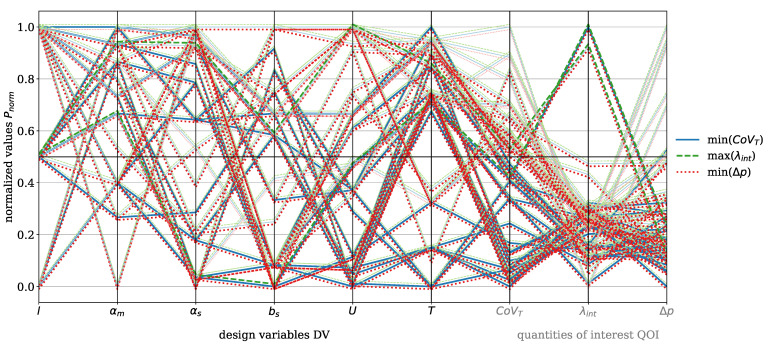
The n=45 evaluations of the moga optimization for the material HDPE and the Saxton element using the DV length *l*, main angle $\alpha_{m}$, sub angle $\alpha_{s}$, perpendicular flight width $b_{s}$, circumferential velocity *U*, and temperature *T*, and the QOI covariance of temperature $CoV_{T}$ and reconstructed interface $\lambda_{int}$ to be maximized, and pressure drop Δp to be minimized, with darker tones indicating values above 0.5 for maximization and below 0.5 for minimization.

The focus now shifts to the Saxton element, beginning with the parallel coordinate plot shown in Figure 9 for the material HDPE. The DVs for this element include length *l*, main angle $\alpha_{m}$, sub angle $\alpha_{s}$, perpendicular flight width $b_{s}$, circumferential velocity *U*, and temperature *T*. The QOI are the covariance of the temperature $CoV_{T}$ and the reconstructed interface $\lambda_{int}$, which are to be maximized, and the pressure drop Δp, which is to be minimized. The color coding and the offset of the data points are applied analogously to those in Figure 7. The figure shows minimal recognizable trends between $CoV_{T}$, ΔpΔp, and $\lambda_{int}$ in the considered design space. Two specific evaluations stand out, characterized by a reconstructed interface value exceeding 0.5, where both the pressure drop and the covariance of temperature are below 0.5. However, these two points do not provide sufficient insight into which geometric parameter predominantly influences the reconstructed interface. Within the analyzed design space, it is evident that $\alpha_{s}>0.5$ and $b_{s}<0.5$ contribute to a pressure drop below 0.5. A sub angle greater than 0.5 reduces the twisting of the secondary channels, allowing the melt to flow more directly in the flow direction. This decreases resistance and can result in lower pressure drop. Furthermore, the covariance of the temperature shows a direct correlation with the pressure drop, because if Δp>0.5 then $CoV_{T}\geq 0.5$, which consequently leads to higher temperature gradients. These conditions are primarily observed for process temperatures below 0.5.

**Figure 10:**
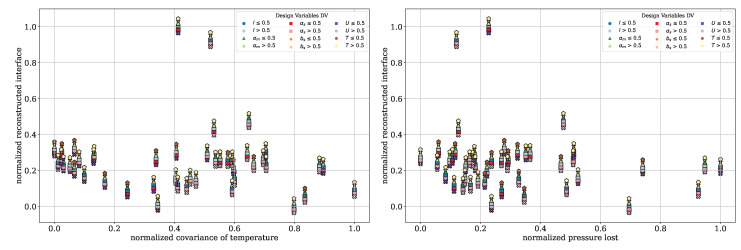
Pareto front for the material HDPE and the Saxton element for the normalized reconstructed interface versus the normalized covariance of temperature (**left**) and the pressure drop (**right**). The length *l*, main angle $\alpha_{m}$, sub angle $\alpha_{s}$, perpendicular flight width $b_{s}$, circumferential velocity *U*, and temperature *T* are the DVs. The marker shapes represents one DV. Dark tones indicate DV values less than 0.5, while light tones represent values greater than 0.5.

Figure 10 illustrates the reconstructed interface plotted against the normalized covariance of temperature (left) and the pressure drop (right). The color coding and marker shapes are consistent with those presented in Figure 8. This view further supports the findings from the plot of parallel coordinates, indicating no visible dependency or contradiction of the reconstructed interface with respect to the other two QOI. The lack of correlation may stem from limitations in the number of particles used and the current implementation of the reconstructed interface calculation. This computation, implemented in *python*, becomes increasingly inefficient as the particle count rises, primarily due to the absence of parallelization. Preliminary investigations reveal that when the particle count exceeds 5000, the computation time becomes excessively long, making the optimization process inefficient. However, reducing the particle count to address computational inefficiency may compromise the reliability and robustness of the reconstructed interface metric. By comparison, Erb utilized $2\times 10^{6}$ particles in his dissertation, with $10^{6}$ particles per cloud, highlighting the potential limitations of the current approach. To calculate the reconstructed interface with such a high number of particles, parallelization of the computation is essential to ensure efficiency and practical usability. However, Figure 10, left illustrates more clearly that $CoV_{T}$ is primarily observed for U>0.5 and T≤0.5. In contrast, Figure 10 (right) shows no distinct dependency of the pressure drop on the operating points (T,U).

With regard to the QOI of the dispersive mixing element, there is only one evolution that represents a compromise of all QOI, dispersive and distributive. The QOI and their corresponding values for DV are listed in Table 5.

### 3.3. Moga Optimization for PP

The analysis now focuses on the material PP. In comparison to HDPE, PP has a lower zero-shear viscosity $\eta_{0}$, a higher density, and an approximately equivalent shear-thinning behavior (see Table 3).

**Figure 11:**
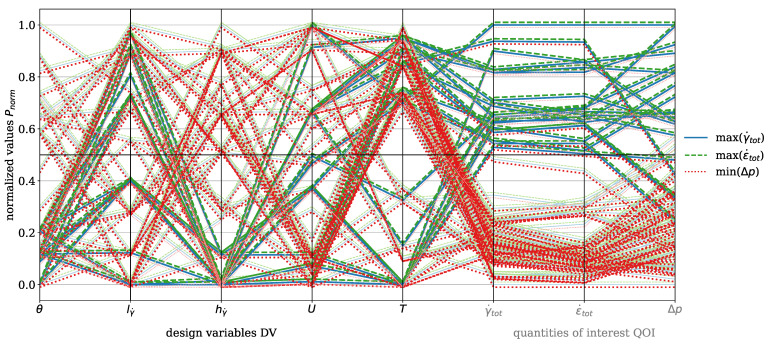
The n=88 evaluations of the moga optimization for the material PP and the dispersive mixing element using the DV wedge angle θ, rectangular slit length $l_{\dot{\gamma }}$ and height $h_{\dot{\gamma }}$, circumferential velocity *U*, and temperature *T*, and the QOI total shear rate ${\dot{\gamma }}_{\mathrm{tot}}$ and total elongation rate ${\dot{\epsilon }}_{\mathrm{tot}}$ to be maximized, and pressure drop Δp to be minimized, with darker tones indicating values above 0.5 for maximization and below 0.5 for minimization.

**Figure A1:**
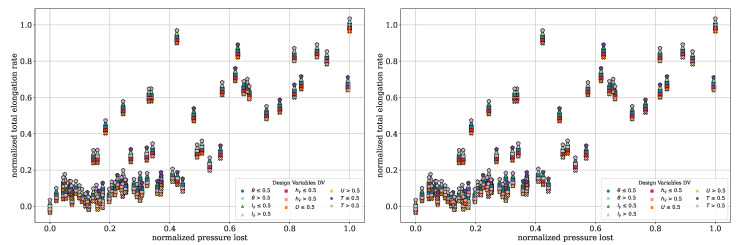
Pareto front for the material PP and the dispersive mixing element.

Figure 11 presents the parallel coordinates plot for the dispersive mixing element with the material PP. The visualization follows the same concept as for HDPE (see Figure 7). The color coding, shifting, line styles, DVs, and QOI remain identical. The number of evaluations is n=88. Figure 11 shows that high deformation rates correlate with increased pressure drop, while low deformation rates result in a reduced pressure drop. Similar to HDPE, high deformation rates are promoted when θ≤0.5 and $h_{\dot{\gamma }}\leq 0.5$. However, the influence of operating conditions is more pronounced for PP. When T>0.5 and U≤0.5, a pressure drop lower than 0.5 is observed. Additionally, three evaluations achieve ${\dot{\gamma }}_{\mathrm{tot}},{\dot{\epsilon }}_{\mathrm{tot}}>0.5$ while maintaining Δp≤0.5. For improved clarity, the Pareto front plots for the material PP for the dispersive mixing element are provided in Appendix C, Figure A1.

**Figure 12:**
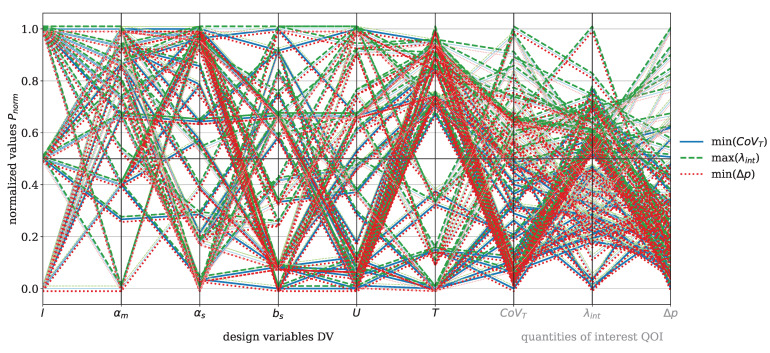
The n=88 evaluations of the moga optimization for the material PP and the Saxton element using the DV length *l*, main angle $\alpha_{m}$, sub angle $\alpha_{s}$, perpendicular flight width $b_{s}$, circumferential velocity *U*, and temperature *T*, and the QOI covariance of temperature $CoV_{T}$ and reconstructed interface $\lambda_{int}$ to be maximized, and pressure drop Δp to be minimized, with darker tones indicating values above 0.5 for maximization and below 0.5 for minimization.

The DV and QOI for the Saxton element with the material PP are shown in Figure 12. The visualization follows the same concept as in Figure 9 for HDPE. A direct correlation between the pressure drop and the covariance of temperature with the operating conditions *T* and *U* is observed. When T>0.5 and U≤0.5, the conditions result in $CoV_{T}\leq 0.5$ and Δp<0.5. Tracing the lines further left to the geometry-related DVs, this state predominantly occurs at $b_{s}\leq 0.5$ and $\alpha_{s}>0.5$. This time, a trend for $\lambda_{\mathrm{int}}$ is also recognizable. Under the listed conditions of $\alpha_{s}$ and $b_{s}$, $\lambda_{\mathrm{int}}>0.5$ stands out more prominently in this plot.

**Figure 13:**
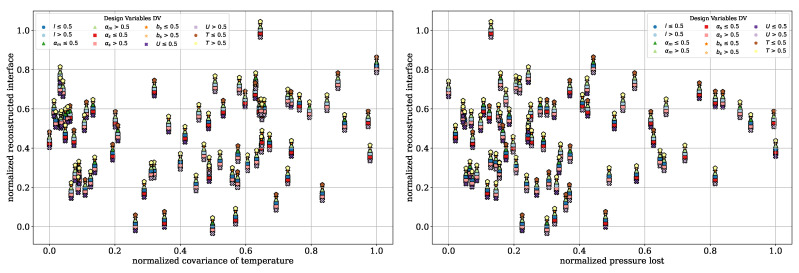
Pareto front for the material PP and the Saxton element for the normalized reconstructed interface versus the normalized covariance of temperature (**left**) and the pressure drop (**right**). The length *l*, main angle $\alpha_{m}$, sub angle $\alpha_{s}$, perpendicular flight width $b_{s}$, circumferential velocity *U*, and temperature *T* are the DVs. The marker shapes represents one DV. Dark tones indicate DV values less than 0.5, while light tones represent values greater than 0.5.

Due to this trend, observable in the parallel coordinate plot, the reconstructed interface is further analyzed in relation to $CoV_{T}$ and Δp, as shown in Figure 13. The concept of the plot representation follows the description provided for Figure 10 in Section 3.2. A significant scattering is observed between $\lambda_{int}$ and $CoV_{T}$ in Figure 13 (left). However, it can be noted that U<0.5 generally corresponds to $CoV_{T}<0.5$, and vice versa. However, a correlation with $\lambda_{int}$ remains unclear. Furthermore, the geometric DVs do not exhibit any discernible correlation, except for the length of the Saxton element, where l<0.5 leads to $\lambda_{int}<0.5$. This is an expected result, as improved mixing can be achieved with a longer mixing path. Similarly, the detailed analysis between $\lambda_{int}$ and Δp in Figure 13 (right) also reveals no direct correlation. The fact that there are no correlations between the reconstructed interface and the DV, $CoV_{T}$ and Δp, as observed in Figure 13, may similarly be attributed to the limitations in the number of particles used and the current implementation of the reconstructed interface calculation. As discussed for HDPE, achieving reliable and efficient computations for the reconstructed interface requires parallelization to manage the large number of particles effectively. With regard to the QOI of the dispersive mixing element, $\lambda_{int}>0.5$, $CoV_{T}\leq 0.5$ and Δp≤0.5 are selected as the best compromises for good mixing efficiency, and their evaluations are listed in Table 5.

### 3.4. Moga Optimization for PLA

The analysis turns to the material PLA. In comparison to HDPE and PP, the zero-shear viscosity of PLA is $\eta_{0,PP}<\eta_{0,PLA}<\eta_{0,HDPE}$ and the density is $\rho_{HDPE}<\rho_{PP}<\rho_{PLA}$. The shear-thinning behavior with c=0.6224 is higher compared to HDPE and PP (see Table 3).

**Figure 14:**
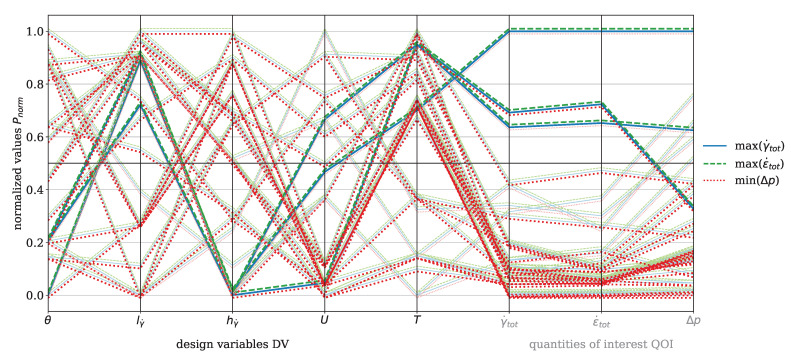
The evaluations of the moga optimization for the material PLA and the dispersive mixing element using the DV wedge angle θ, rectangular slit length $l_{\dot{\gamma }}$ and height $h_{\dot{\gamma }}$, circumferential velocity *U*, and temperature *T*, and the QOI total shear rate ${\dot{\gamma }}_{\mathrm{tot}}$ and total elongation rate ${\dot{\epsilon }}_{\mathrm{tot}}$ to be maximized, and pressure drop Δp to be minimized, with darker tones indicating values above 0.5 for maximization and below 0.5 for minimization.

Figure 14 shows the parallel coordinates plot for the dispersive mixing element with the material PLA. The visualization follows the same approach as for HDPE (see Figure 7) with consistent color coding, data shifting, line styles, DVs, and QOI. The number of evaluations is n=32. Similar to HDPE and PP, high deformation rates are observed when θ≤0.5 and $h_{\dot{\gamma }}\leq 0.5$. For PLA, one moga evolution achieves a state where ${\dot{\gamma }}_{\mathrm{tot}}>0.5$, ${\dot{\epsilon }}_{\mathrm{tot}}>0.5$, and Δp≤0.5. This condition is associated with T>0.5, U>0.5, $h_{\dot{\gamma }}\leq 0.5$, $l_{\dot{\gamma }}>0.5$, and θ≤0.5. In general, high temperatures and low circumferential velocities result in Δp<0.5.

**Figure 15:**
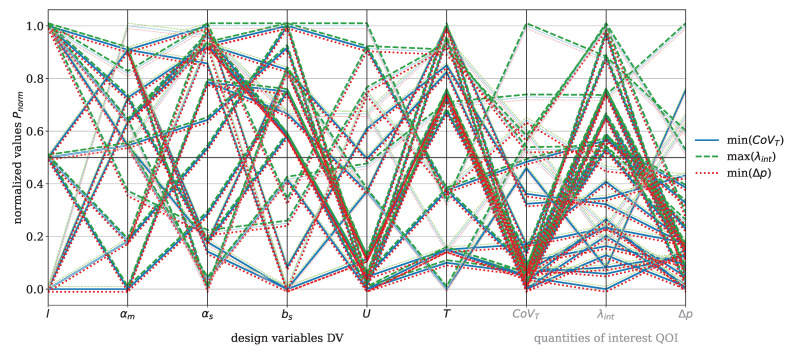
The evaluations of the moga optimization for the material PLA and the Saxton element using the DV length *l*, main angle $\alpha_{m}$, sub angle $\alpha_{s}$, perpendicular flight width $b_{s}$, circumferential velocity *U*, and temperature *T*, and the QOI covariance of temperature $CoV_{T}$ and reconstructed interface $\lambda_{int}$ to be maximized, and pressure drop Δp to be minimized, with darker tones indicating values above 0.5 for maximization and below 0.5 for minimization.

**Figure A2:**
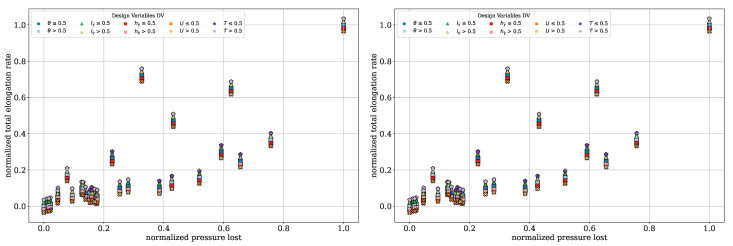
Pareto front for the material PLA and the dispersive mixing element.

**Figure A3:**
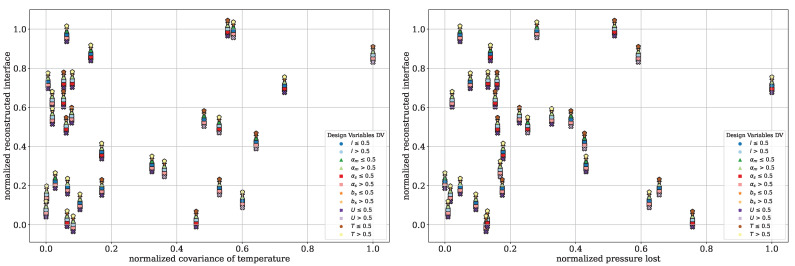
Pareto front for the material PLA and the Saxton element.

Figure 15 presents the DVs and QOI for the Saxton element with PLA, following the same structure as in Figure 9. A direct correlation between Δp and $CoV_{T}$ with *T* and *U* is visible. When T>0.5 and U≤0.5, Δp≤0.5 and $CoV_{T}\leq 0.5$ are observed. For PLA, there is also a correlation between $\lambda_{int}$ and the other QOI. Evaluations with Δp≤0.5 and $\lambda_{int}>0.5$ also show $CoV_{T}\leq 0.5$. However, evaluations with $\lambda_{int}>0.5$ show no significant influence from the geometric DVs, except for the element length *l*, where l>0.5 corresponds to $\lambda_{int}>0.5$. With regard to the QOI of the dispersive mixing element, the evaluations achieving $\lambda_{int}>0.5$, $CoV_{T}\leq 0.5$, and Δp≤0.5 are selected as compromises for high mixing efficiency. These evaluations are listed in Table 5. The Pareto front plots for PLA for both mixing elements are included in Appendix C, Figure A2 (dispersive), and Figure A3 (distributive).

### 3.5. Moga Optimization for PS

The analysis now focuses on the material PS. In comparison to HDPE, PP, and PLA, the zero-shear viscosity of PS is $\eta_{0,PP}<\eta_{0,PLA}<\eta_{0,PS}<\eta_{0,HDPE}$ and the density is $\rho_{HDPE}<\rho_{PS}<\rho_{PP}<\rho_{PLA}$. With c=0.685, PS exhibits the most pronounced shear-thinning behavior compared to the other materials (see Table 3).

**Figure 16:**
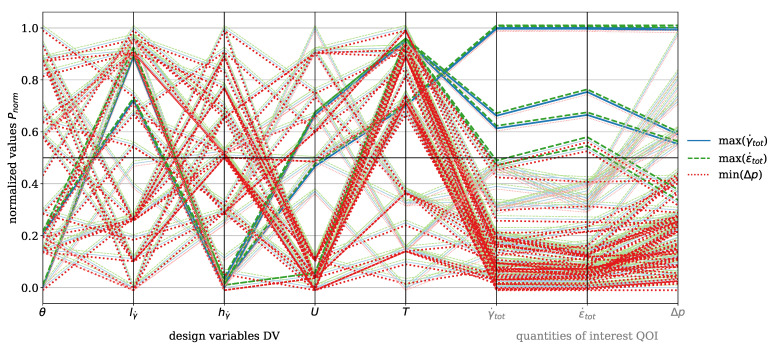
The evaluations of the moga optimization for the material PS and the dispersive mixing element using the DV wedge angle θ, rectangular slit length $l_{\dot{\gamma }}$ and height $h_{\dot{\gamma }}$, circumferential velocity *U*, and temperature *T*, and the QOI total shear rate ${\dot{\gamma }}_{\mathrm{tot}}$ and total elongation rate ${\dot{\epsilon }}_{\mathrm{tot}}$ to be maximized, and pressure drop Δp to be minimized, with darker tones indicating values above 0.5 for maximization and below 0.5 for minimization.

Figure 16 presents the parallel coordinates plot for the dispersive mixing element with the material PS. The visualization approach remains consistent with HDPE (see Figure 7). The number of evaluations is n=77. A pressure drop below 0.5 is achieved for T<0.5, U≤0.5, and predominantly when $h_{\dot{\gamma }}>0.5$. Evaluations where $h_{\dot{\gamma }}\leq 0.5$ and Δp≤0.5 occur are correlated with T>0.5. A condition where T≤0.5 and Δp≤0.5 is only met when U>0.5. However, for the dispersive mixing element, no evaluations satisfy the criteria of ${\dot{\gamma }}_{\mathrm{tot}}>0.5$, ${\dot{\epsilon }}_{\mathrm{tot}}>0.5$, and Δp≤0.5. Only when the threshold for Δp is reduced to 0.4 do two compromise solutions emerge. High deformation rates are again observed for $h_{\dot{\gamma }}\leq 0.5$, $l_{\dot{\gamma }}>0.5$, and θ≤0.5.

**Figure 17:**
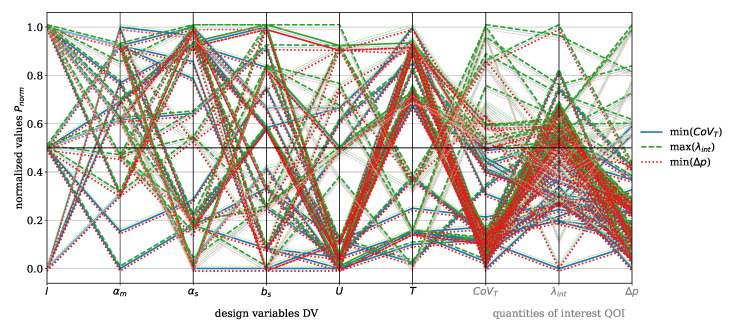
The evaluations of the moga optimization for the material PS and the Saxton element using the DV length *l*, main angle $\alpha_{m}$, sub angle $\alpha_{s}$, perpendicular flight width $b_{s}$, circumferential velocity *U*, and temperature *T*, and the QOI covariance of temperature $CoV_{T}$ and reconstructed interface $\lambda_{int}$ to be maximized, and pressure drop Δp to be minimized, with darker tones indicating values above 0.5 for maximization and below 0.5 for minimization.

**Figure A4:**
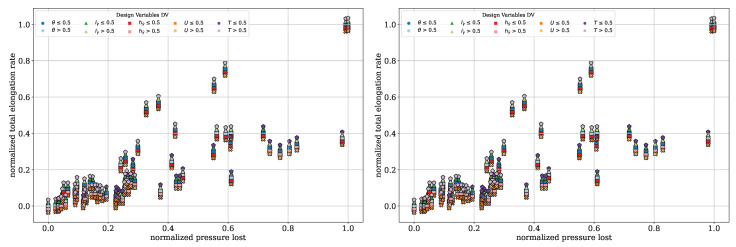
Pareto front for the material PS and the dispersive mixing element.

**Figure A5:**
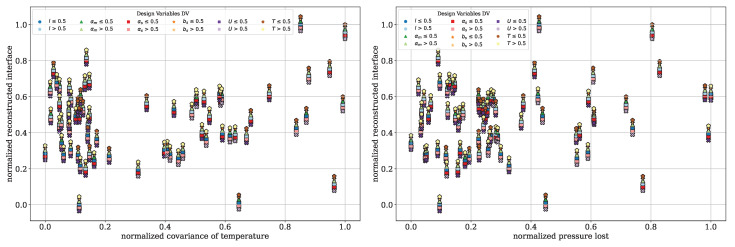
Pareto front for the material PS and the Saxton element.

The DV and QOI for the Saxton element with the material PS are shown in Figure 17. The visualization remains consistent with HDPE (see Figure 9). Again, the trend can be observed that $CoV_{T}\leq 0.5$ and Δp≤0.5 primarily result from the operating conditions T>0.5 and U≤0.5. Otherwise, no recognizable correlation between reconstructed interface greater than 0.5 and the geometric DVs is observed for the material PS. With regard to the QOI of the dispersive mixing element, $\lambda_{int}>0.4$, $CoV_{T}\leq 0.4$ and Δp≤0.4 are selected as the best compromises for good mixing efficiency and their evaluations listed in Table 5. For clarity, the Pareto front plots for the material PS for both mixing elements are provided in Appendix C, Figure A4 (dispersive), and Figure A5 (distributive).

### 3.6. Material Comparison for Performance and Stability in CFD Simulations

To provide a clear comparison of the materials HDPE, PP, PLA, and PS, Table 5 lists the selected evaluations, showing the corresponding DVs and QOI in their respective units. These evaluations represent the best compromise solutions identified in previous sections, fulfilling the criteria of achieving high deformation rates and reconstructed interface values while maintaining low covariance of temperature and pressure drop.

In the considered temperature range 180 °C <T< 220 °C, significant differences in performance emerge among the materials. The material PP exhibits the lowest Δp, while HDPE experiences the highest. This indicates that HDPE has a higher viscosity within this temperature range, resulting in greater resistance to flow, followed by PLA. PS achieves a moderate Δp, falling between PP and PLA. These observations highlight the impact of viscosity on flow resistance and underscore the role of material properties in determining DV and QOI.

The Carreau model parameters—zero-shear viscosity *a*, relaxation time *b*, and shear-thinning behavior *c*—play a crucial role in the material flow behavior. These parameters influence simulation performance and stability, making them key factors in the simulation process.

The complexity of the Saxton geometry, with its numerous degrees of freedom, posed significant challenges during the optimization process. Frequent errors were encountered in *Onshape* and *OpenFOAM*, particularly under certain geometrical DV. The high degree of geometric complexity prevents the derivation of a general geometric function capable of producing valid geometries across the entire design space. To mitigate this, the implemented error detection in the automation script excludes DV variations where the generation of *stl* files is not possible. Notably, this issue was independent of the material being evaluated.

Despite successful geometry export, simulation instabilities often arose, especially for HDPE. These instabilities were more frequent during LPT simulations in post-processing, even when a converged velocity field for the Saxton geometry was achieved.

The success rates of the moga evaluations varied significantly among the materials. For HDPE, of the total n=255 evaluations, only 45 were usable for analysis, but 132 evaluations were successful for the dispersive element. In contrast, PP achieved n=185 evaluations, of which 88 were analyzed successfully, showing equal performance for both dispersive and Saxton elements. PLA had n=63 evaluations, with 32 usable results; excluding the Saxton element, this number would have been slightly higher at 34. For PS, n=92 evaluations were conducted, with 77 analyzed successfully, again demonstrating equal performance for both element types.

These results indicate that HDPE, due to its high zero-shear viscosity and longer relaxation time, introduces more instability in CFD simulations. This is likely caused by pronounced viscosity gradients within the fluid domain. PLA follows HDPE in instability but exhibits a less pronounced shear-thinning behavior, resulting in more stable LPT simulations.

Beyond the issue of an insufficient number of particles compromising the reliability and robustness of the reconstructed interface, it is often observed with HDPE that no particles reach the outlet in the LPT simulations within the specified end time. As a result, the reconstructed interface could not be calculated, which explains the significant discrepancy between the evaluations performed and those ultimately analyzed for HDPE. These findings underscore the critical influence of the Carreau parameters on simulation stability and the trade-offs required for optimizing mixing performance.

Further comparisons of the materials in terms of simulation instability or mixing performance remain a challenge, as the DVs selected with the moga optimizer are not identical for all materials. Instead, the results should be interpreted as material-specific trade-offs within the given design space.

## 4. Conclusions and Outlook

A successful automated optimization tool for mixing elements has been developed and tested for the materials HDPE, PP, PLA, and PS. This tool efficiently identifies optimal trade-offs, providing the QOI alongside the appropriate geometries of the mixing elements and operating conditions, while accounting for material-specific properties.

The pattern integrated into the CAD software for cutting the surface of the mixing elements has proven to be highly effective for automation. This approach ensured that almost equilateral triangles are created in the *stl* files, resulting in smooth processing during meshing. The four-stage simulation approach, with increasing complexity in the viscosity model, proved to be highly efficient. Following its implementation, the simulations exhibited stability and achieved convergence within the fluid domains of both mixing elements. Together, the pattern and the four-stage simulation framework facilitated full automation. Additionally, the chosen operating parameters, circumferential velocity of the screw *U* and temperature *T*, proved to be effective DVs for optimizing the mixing element.

The selected software has proven to be suitable. As they are operating system-independent, i.e., guarantee broad usability, and are freely available, the interface between the various programs can be bridged. The framework of *openFoam*^®^is highly flexible, allowing modifications to implement custom material models or post-processing utilities. In addition, it is possible to use all three software with open-source scripting languages such as *Bash* and *Python*, which results in a high degree of automation.

The parameterized geometry of the dispersive mixing element demonstrated high suitability, offering a large design space with stable simulations despite changes in geometry. The chosen parametrization revealed clear correlations between the DVs and QOI, such as lower rectangular slit height *h* and higher slit length *l* favoring higher deformation rates, thereby enhancing mixing efficiency. Additionally, the chosen QOI, including the total shear rate ${\dot{\gamma }}_{tot}$ and elongation rate ${\dot{\epsilon }}_{tot}$, proved to be effective quantitative metrics for evaluating the system in terms of droplet breakup potential. An optimum wedge-angle position less than 0.2 is crucial for a pronounced elongational flow and depends on the flow behavior of the material.

The Saxton element was also successfully investigated, but its higher complexity posed challenges in establishing correlations between the DVs and QOI. Simulations that converged did not show clear correlations between the selected DVs and QOI. However, analyzing *U* and *T* in relation to pressure drop Δp and covariance of temperature $CoV_{T}$ revealed that lower *U* and higher *T* tend to minimize both Δp and $CoV_{T}$. No correlations could be identified between the reconstructed interface $\lambda_{int}$ and the chosen geometry DVs in this study. Additionally, the success of LPT simulations was highly dependent on the geometry DVs, and the current implementation of $\lambda_{\mathrm{int}}$ is associated with high computational times.

Moreover, material properties strongly influence DV and QOI results. HDPE, with its high Carreau model parameter *a* and *b*, poses significant challenges for stable CFD simulations, especially for the Saxton element. Materials such as PP and PS demonstrate better performance due to their moderate zero-shear viscosity and relaxation time in the considered temperature range.

Furthermore, it must be acknowledged that certain assumptions were introduced, including the simplifications to a single-phase simulation. Nevertheless, the shear and strain rates provide information about the deformation behavior of the melt within the mixing elements. This offers a reasonable approximation for applying the optimization tool in real-world processing conditions. This is, higher rates increase the probability of droplet break-up. To prevent material degradation, prior knowledge of the material’s tolerance to deformation is required. If this is known, a suitable compromise can be chosen, and the corresponding geometry can be manufactured to ensure a homogeneous melt at the given screw circumferential velocity and temperature during converting.

Future work should change the DVs of the Saxton element and reduce the degree of freedom and thus the design space. The use of *openFoam*^®^infrastructure and proper implementation can enable parallel computation of the reconstructed interface, allowing for efficient analysis with more particles and improved stability and reliability. Additionally, future optimization should include residence time as an additional QOI to prevent material degradation and enhance LPT efficiency. Favoring DVs that minimize residence time will identify cases where particles reach the outlet in an acceptable time. These steps will enhance the accuracy and robustness of the simulations, leading to better optimization of mixing performance for various shear-thinning materials. In addition, further work will focus on the validation of the optimization tool to assess its accuracy and applicability. This will include an experimental comparison with real extrusion processes.

## Figures and Tables

**Table 1:** Left: The characteristic parameters of the Saxton element and and their dependency on the DVs (perpendicular flight width $b_{s}$, helix angle of the main $\alpha_{m}$ and side $\alpha_{s}$ channel, length *L* with l∈Z), where $D_{i}$ denotes the screw and $D_{a}$ the barrel diameters of an SSE. The geometry is split into one main channel (i=m) and six side channels (i=s). Right: The characteristic parameters of the dispersive mixing element and their dependency on the DVs (angle of the wedge θ, height $h_{\dot{\gamma }}$, and length $l_{\dot{\gamma }}$ of the rectangular slit). Bottom: The DVs listed for the respective mixing element.

Saxton	Main Channel	Side Channel	Dispersive	
Flight width $e_{i}$	$e_{m}=8mm$	$e_{s}=f\left(b_{s},\alpha_{s},\alpha_{m}\right)$	wedge length	$l_{\dot{\varepsilon }}$ = 6 mm
Perpendicular flight width $b_{i}$	$b_{m}=f\left(\alpha_{m}\right)$	-	slit orientation	α=32.14°
Helix pitch $t_{i}$	$t_{m}=f\left(\alpha_{m}\right)$	$t_{s}=f\left(\alpha_{s}\right)$	wedge height	$h_{\dot{\varepsilon }}=f\left(\theta ,l_{\dot{\varepsilon }}\right)$
Channel depth $h_{i}$	$h_{m}=h_{s}=\frac{1}{2}\left(D_{a}-D_{i}\right)$		screw diameter	$D_{i}=f\left(\theta ,h_{\dot{\varepsilon }}\right)$
Number of channels $N_{i}$	$N_{m}=1$	$N_{s}=6$	A,B,C,β	$f(\theta ,l_{\dot{\varepsilon }},l_{\dot{\gamma }})$
Design variables DV	$b_{s},\alpha_{m},\alpha_{s},L$		$\theta ,l_{\dot{\gamma }},h_{\dot{\gamma }}$	

**Table 2:** Boundary conditions for each flow parameter $\vec{u},p,T$ at the *patches* of the fluid domain within the single reference frame (SRF) for both (dispersive and Saxton) mixing elements. The Saxton inlet is split in two (left, right). The initial inlet velocity value $\vec{u}$ of the inlet and cylinder is determined by a given mass rate $\dot{m}$, the inlet area *A*, and the flow density $\rho_{F}$. The initial inlet $T_{disp}$-profile of the Saxton is the result of the dispersive mixing element simulation.

*Patch*	Element	Boundary Conditions					
	$\vec{u}$ ** in m/s**		p **in Bar**		T **in K**
cylinder	both	SRFVelocity	$\frac{\dot{m}}{\rho_{F}\;\cdot \;A}$	Neumann	-	Dirichlet	DV
screw	both	Dirichlet	0	Neumann	-	Dirichlet	DV
inlet	dispersive	SRFVelocity	$\frac{\dot{m}}{\rho_{F}\;\cdot \;A}$	Neumann	-	Dirichlet	DV
inlet(left,right)	Saxton						$T_{\mathrm{disp}}$
outlet	both	Neumann	-	Dirichlet	0	Neumann	-

**Table 3:** Carreau–Arrhenius parameters a,b,c, the activation energy *E*, the reference temperature $T_{0}$, the specific heat capacity $c_{p}$, and the thermal conductivity λ of polystyrene (PS), polypropylene (PP), polylactide (PLA) and high-density polyethylene (HDPE) [16,24].

Material	ρ/$kg/m^{-3}$	*a*/Pas	*b*/s	*c*/-	*E*/$J/{\mathrm{mol}}^{-1}$	$T_{0}$/K	$c_{p}$/$m^{2}s^{-2}K^{-1}$	λ/$kgms^{-2}K^{-1}$
PS	781	3303	0.1923	0.6855	98239	493.15	2082	0.1241
PP	780	1064	0.1203	0.549	45733	493.15	2780	0.16
PLA	1118	1703	0.024	0.6224	73654	473.15	2160	0.16
HDPE	750	195812	16.7459	0.58566	19519.7	473.15	2770	0.2597

**Table 4:** Top: Design space. On the left: The DV of Saxton element with its main channel i=m and sub channel i=s and the constraints. On the right: The DV of the dispersive mixing element and the constraints, as well as the DV of the operation points (U,T). Bottom: Quantities of interest. Maximize the shear ${\dot{\gamma }}_{tot}$ and elongation rate ${\dot{\epsilon }}_{tot}$ and the reconstructed interface $\lambda_{int}$. Minimize the pressure drop Δp and covariance of the temperature $CoV_{T}$.

	Saxton Element			Dispersive Element		
**Design Variables DV**	**Main Channel**	**Side Channel**	**Unit**	**DV**		**Unit**
helix angle $\alpha_{i}$	$5\leq \alpha_{m}\leq 20$	$20\leq \alpha_{s}\leq 80$	°	wedge angle	6≤θ≤45	°
perpendicular flight width $b_{i}$	$\left(b_{m}=8\right)$	$6\leq b_{s}\leq 12$	mm	rectangular slit height	$0.6\leq h_{\dot{\gamma }}\leq 2$	mm
length l∈Z	1≤l≤3		-	rectangular slit length	$4\leq l_{\dot{\gamma }}\leq 8$	mm
circumferential velocity *U*	<U<		$\min^{-1}$	Temperature *T*	180<T<220	°C
Quantities of interest QOI	max(${\dot{\gamma }}_{tot},{\dot{\epsilon }}_{tot},\lambda_{int}$)			min(Δp, $CoV_{T}$)		

**Table 5:** Selected evaluations for HDPE, PP, PS, and PLA. Left: DV of both mixing elements such as temperature *T*, circumferential velocity *U*; dispersive: wedge angle θ, rectangular slit length $l_{\dot{\gamma }}$ and height $h_{\dot{\gamma }}$; Saxton: length *l*, main angle $\alpha_{m}$, sub angle $\alpha_{s}$, perpendicular flight width $b_{s}$. Right: QOI pressure drop Δp of dispersive such as total shear rate ${\dot{\gamma }}_{\mathrm{tot}}$ and elongation rate ${\dot{\epsilon }}_{\mathrm{tot}}$ and Saxton elements, such as covariance of temperature $CoV_{T}$ and reconstructed interface $\lambda_{int}$.

	Design Variable DV									Quantity of Interest QOI				
			Dispersive			Saxton					Dispersive		Saxton	
Material	*T* in °C	*U* in min^−1^	*θ* in °	$h_{\dot{\gamma }}$ in μm	$l_{\dot{\gamma }}$ in μm	*l* in -	*α_m_* in °	*α_s_* in °	*b_s_* in mm	Δp in bar	${\dot{\gamma }}_{\mathrm{tot}}$ in s^−1^	${\dot{\varepsilon }}_{\mathrm{tot}}$ in s^−1^	${\mathrm{CoV}}_{T}$ in -	$\lambda_{\mathrm{int}}$ in -
											$\times 10^{4}$	$\times 10^{4}$	$\times 10^{-2}$	
HDPE	186	82	12	104	755	1	11	55	6.5	596.61	483.0	83.8	0.62	11.55
PP	216	82	15	63	682	2	11	66	6.5	105.80	402.6	83.7	0.07	17.35
	214	56	8	63	682	3	18	77	10.0	125.30	593.7	120.8	0.04	36.83
	180	100	12	78	558	1	16	50	10.0	138.19	371.4	71.0	0.14	3.96
	210	56	15	63	558	3	15	68	10.0	84.38	379.4	75.6	0.04	39.56
	214	82	15	63	682	3	19	66	6.5	104.03	402.5	83.5	0.06	12.49
PLA	218	71	15	63	755	3	16	77	9.5	202.96	458.7	97.1	0.08	22.83
PS	218	71	15	63	755	3	16	77	9.5	121.95	375.1	78.4	0.10	28.16
	218	71	15	63	682	1	20	77	10.0	112.87	363.9	74.3	0.13	14.31

## Data Availability

The data presented in this study are available on request from the corresponding author due to ongoing analysis and further refinement of the dataset.

## Abbreviations

    The following abbreviations are used in this manuscript:

SSE	Single-screw extrusion
CFD	Computational fluid dynamics
DoE	Design of experiment
CAD	Computer-aided design
GA	Genetic algorithm
MOGA	Multi-objective genetic algorithm
OPT	Optimization
DV	Design variable
DP	Design point
SOE	system of equation
LPT	Lagrangian particle simulation

## Appendix A. Automated Optimization for a Dispersive and Distributive Mixing Element

**Algorithm A1:** First: Extract and configure *stl* files from *onshape*. Second: Pre-processing, running simulation and post-processing of dispersive and Saxton element in *openFoam*.

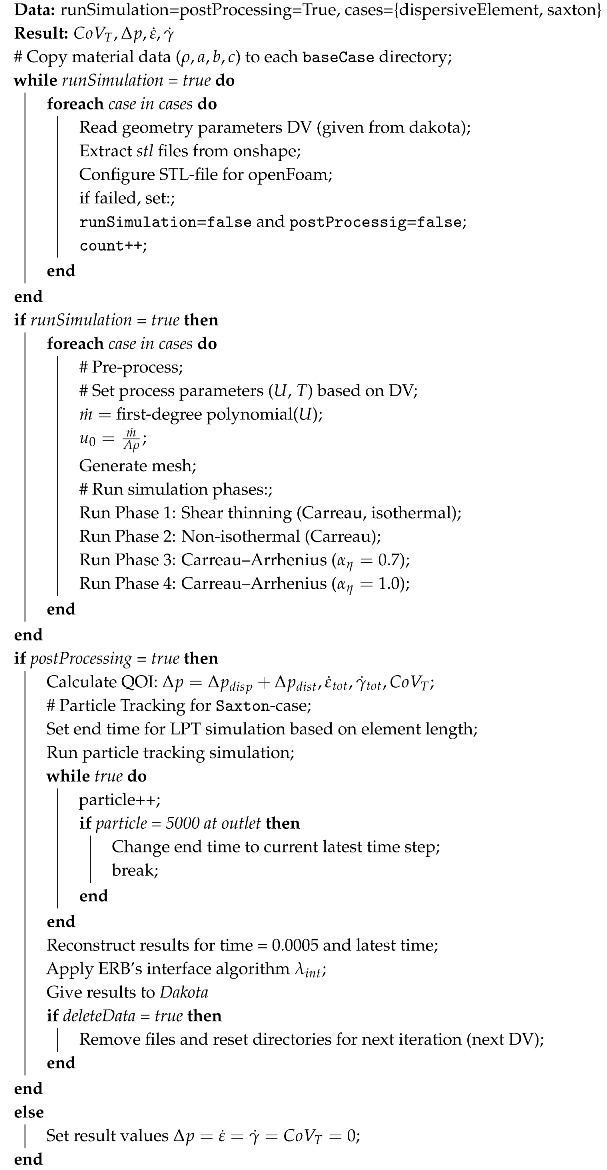

## Appendix B. Material Specifications

**Table A1:** Materials: polystyrene (PS), polypropylene (PP), polylactide (PLA) and high-density polyethylene (HDPE) [16,24].

Material	Manufactures	Brand Name	Polymer
PS	INEOS Styrolution Group GmbH	Styrolution PS 165N/L	polystyrene
PP	Borealis AG	RE420MO	polypropylene
PLA	NatureWorks LLC	Ingeo 7001D	polyactide
HDPE	LyondellBasell Industries	Hostalen ACP 5331 A	high-density polyethylene

## Appendix C. Plots of the Pareto Fronts of Different Materials and Mixing Elements

The Pareto fronts for PP, PLA, and PS for the dispersive mixing elements show total elongation rate (left) and shear rate (right) versus normalized pressure drop. Design variables (DVs) include wedge angle θ, rectangular length $l_{\dot{\gamma }}$ and height slit $h_{\dot{\gamma }}$, circumferential velocity *U*, and temperature *T*. For PLA and PS, the Pareto fronts for the Saxton element show normalized reconstructed interface versus normalized covariance of temperature (left) and pressure drop (right). The DVs are length *l*, main angle $\alpha_{m}$, sub angle $\alpha_{s}$, perpendicular flight width $b_{s}$, circumferential velocity *U*, and temperature *T*. Marker shapes represent DVs, with dark tones for values below 0.5 and light tones for values above 0.5.

## References

[1] Bonten C. (2019). Plastics Technology: Introduction and Fundamentals.

[2] Otero Navas I., Kamkar M., Arjmand M., Sundararaj U. (2021). Morphology Evolution, Molecular Simulation, Electrical Properties, and Rheology of Carbon Nanotube/Polypropylene/Polystyrene Blend Nanocomposites: Effect of Molecular Interaction between Styrene-Butadiene Block Copolymer and Carbon Nanotube. Polymers.

[3] Li H., Sundararaj U. (2009). Morphology development of polymer blends in extruder: The effects of compatibilization and rotation rate. Macromol. Chem. Phys..

[4] Karam H.J., Bellinger J.C. (1968). Deformation and Breakup of Liquid Droplets in a Simple Shear Field. Ind. Eng. Chem. Fundam..

[5] Geiger K., Grünschloss E., Martin G., Platz E. (2013). Scher- und Dehndeformationen von Kunststoffschmelzen im Keilspalt. Chem. Ing. Tech..

[6] Chella R., Ottino J. (1985). Stretching in some classes of fluid motions and asymptotic mixing efficiencies as a measure of flow classification. Arch. Ration. Mech. Anal..

[7] Boss J., Poland W. (1986). Evaluation of the homogeneity degree of a mixture. Bulk Solids Handl..

[8] Phelps J.H., Tucker C.L. (2006). Lagrangian particle calculations of distributive mixing: Limitations and applications. Chem. Eng. Sci..

[9] Erb T. (2018). Simulationsgestützte Optimierung dynamischer Mischer für Hochleistungsextruder. PhD Dissertation.

[10] Delaunay B., Spherevide S. (1934). A la memoire de Georges Voronoi. Bull. L’Académie Sci. L’URSS Cl. Sci. Math. Nat..

[11] Eusterholz S., Elgeti S. (2019). Shape Optimization in Polymer Extrusion Using Shear-Thinning Fluids.

[12] Janßen M. (2022). Automatisierte Optimierung von dynamischen Mischelementen. Zeitschrift Kunststofftechnik.

[13] Adams B.M., Bohnhoff W.J., Dalbey K.R., Ebeida M.S., Eddy J.P., Eldred M.S., Hooper R.W., Hough P.D., Hu K.T., Jakeman J.D. (2020). Dakota, A Multilevel Parallel Object-Oriented Framework for Design Optimization, Parameter Estimation, Uncertainty Quantification, and Sensitivity Analysis.

[14] David E. (1989). Golderg Genetic Algorithms in Search Optimization and Machine Learning.

[15] Haldar A., Mahadevan S. (2000). Probability, Reliability and Statistical Methods in Engineering Design.

[16] Celik O. (2018). Neuartiges Simulationsmodell zur Vorhersage der Prozessinduzierten Morphologieausbildung in Heterogenen Kunststoffblends. Ph.D. Dissertation.

[17] Ferziger J., Perić M., Street R. (2020). Computational Methods for Fluid Dynamics.

[18] Hensen F., Burkhardt U. (1989). Handbuch der Kunststoff-Extrusionstechnik: Grundlagen Bd. 1.

[19] Bird R., Stewart W., Lightfoot E. (2006). Transport Phenomena.

[20] Osswald T., Rudolph N. (2015). Polymer Rheology: Fundamentals and Applications.

[21] Wilhelm D. (2015). Rotating flow simulations with OpenFOAM. Int. J. Aeronaut. Sci. Aerosp. Res..

[22] Greenshields C., Weller H. (2022). Notes on Computational Fluid Dynamics: General Principles.

[23] Juretić D.F. (2015). cfMesh User Guide.

[24] Kettemann J. (2022). Simulation der Strömungen von Kunststoffschmelzen in Extrusionsprozessen mithilfe einer Immersed-Boundary-Methode. Ph.D. Dissertation.

